# Network Pharmacology Study to Reveal the Potentiality of a Methanol Extract of *Caesalpinia sappan* L. Wood against Type-2 Diabetes Mellitus

**DOI:** 10.3390/life12020277

**Published:** 2022-02-13

**Authors:** Md. Adnan, Byeong-Bae Jeon, Md. Helal Uddin Chowdhury, Ki-Kwang Oh, Tuhin Das, Md. Nazim Uddin Chy, Dong-Ha Cho

**Affiliations:** 1 Department of Bio-Health Convergence, College of Biomedical Science, Kangwon National University, Chuncheon 24341, Korea; mdadnan@kangwon.ac.kr (M.A.); bbjeon@hffaroma.com (B.-B.J.); nivirna07@kangwon.ac.kr (K.-K.O.); 2 Ethnobotany and Pharmacognosy Lab, Department of Botany, University of Chittagong, Chattogram 4331, Bangladesh; helaluddinchowdhurycu@gmail.com; 3 Department of Microbiology, University of Chittagong, Chattogram 4331, Bangladesh; tuhin.mbio@gmail.com; 4 Department of Pharmacy, International Islamic University Chittagong, Chittagong 4318, Bangladesh; nazim107282@gmail.com

**Keywords:** *Caesalpinia sappan* L., T2DM, PPAR signaling pathway, fisetin tetramethyl ether, network pharmacology

## Abstract

*Caesalpinia sappan* L. (CS) is widely used to treat diabetic complications in south-east Asia, specifically in traditional Chinese medicine. This study intends to explain the molecular mechanism of how chemical constituents of CS interrelate with different signaling pathways and receptors involved in T2DM. GC-MS was employed to identify the chemical compounds from the methanol extract of CS wood (MECSW). Lipinski’s rule of five was applied, and 33 bioactive constituents have been screened from the CS extract. After that, 124 common targets and 26 compounds associated with T2DM were identified by mining several public databases. Protein–protein interactions and compound-target network were constructed using the STRING database and Cytoscape tool. Protein–protein interactions were identified in 121 interconnected nodes active in T2DM and peroxisome proliferator-activated receptor gamma (PPARG) as key target receptors. Furthermore, pathway compound target (PCT) analysis using the merger algorithm plugin of Cytoscape revealed 121 nodes from common T2DM targets, 33 nodes from MECSW compounds and 9 nodes of the KEGG pathway. Moreover, network topology analysis determined “Fisetin tetramethyl ether” as the key chemical compound. The DAVID online tool determined seven signaling receptors, among which PPARG was found most significant in T2DM progression. Gene ontology and KEGG pathway analysis implied the involvement of nine pathways, and the peroxisome proliferator-activated receptor (PPAR) pathway was selected as the hub signaling pathway. Finally, molecular docking and quantum chemistry analysis confirmed the strong binding affinity and reactive chemical nature of fisetin tetramethyl ether with target receptors exceeding that of the conventional drug (metformin), PPARs agonist (rosiglitazone) and co-crystallized ligands, indicating that fisetin could be a potential drug of choice in T2DM management. This study depicts the interrelationship of the bioactive compounds of MECSW with the T2DM-associated signaling pathways and target receptors. It also proposes a more pharmaceutically effective substance, fisetin tetramethyl ether, over the standard drug that activates PPARG protein in the PPAR signaling pathway of T2DM.

## 1. Introduction

Type-2 diabetes mellitus (T2DM) is a global epidemic attributed to the dysregulation of carbohydrate, lipid, and protein metabolism resulting from impaired insulin secretion, insulin resistance, or a combination of both. According to the International Diabetes Federation (IDF) report in 2021, one in ten adults (20 to 79 years) has diabetes mellitus. The IDF also estimated this rate to rise to 643 million by 2030 and 783 million by 2045. Such a significant increase will come from the economic transition from low-income to middle-income levels [1]. 

T2DM is a complex chronic disease involving genetic predisposition, environmental and behavioral risk factors [2,3,4]. Upon the dysfunction of feedback loops between insulin action and insulin secretion, insulin-sensitive tissues, such as liver, muscle, and the adipose tissue, are affected, resulting in abnormal insulin secretion by pancreatic islet β cells and abnormal glucose levels in the blood; this impaired insulin secretion comes forth as rooted sake of T2DM [5]. Insulin resistance causes type-2 diabetes, which mediates increased glucose synthesis in the liver and decreases glucose intake in muscle and adipose tissue at a certain insulin level. Moreover, chronic inflammation leads to impaired control of blood glucose levels, resulting in vascular complications [6]. Likewise, β-cell dysfunction causes reduced insulin release resulting in blood glucose homeostasis failure [7] and the accumulation of reactive oxygen species (ROS) in β cells, also accountable for insulin deficiency through immutable damage in mitochondria [8]. Individuals who suffer from this noteworthy metabolic condition are at a greater risk of mortality from myocardial disease, renal disease, virus-related sickness, and respiratory diseases, among other causes [9,10,11].

Pharmacologically significant agents, such as biguanides, sulfonylureas, meglitinides, thiazolidinediones, alpha-glucosidase inhibitors, incretin-based therapies, dipeptidyl-peptidase IV inhibitors, insulin analogs, and bromocriptine, have been reported as potential for T2DM patients in clinical scenarios [9,12,13,14,15]. Several oral anti-diabetic drugs, including metformin, glimepiride, repaglinide, pioglitazone, sitagliptin, and acarbose, have been used frequently as therapeutic agents. However, these drugs often create side effects, for instance, lactic acidosis, hypoglycemia, anorexia, nausea, dyspeptic episodes, impaired renal function, and other gastrointestinal issues [16,17,18]. While synthetic drugs pose some adverse effects in the patient, a wide range of plant-derived compounds are showing promising outcomes in managing T2DM [19]. That is why plant-derived metabolites have been way ahead of synthetic ones in the quest for a safer therapeutic agent of T2DM. Since different medicinal plants are reported and widely used to manage diabetes as per traditional practice in the south Asian region, this study aims to draw some insight into the selected plant’s anti-diabetic activity, *Caesalpinia sappan*, which has been reported as having anti-diabetic activity in several recent studies [20,21,22,23,24].

*Caesalpinia sappan* L. is also known as *Biancaea sappan* (L.) Tod and belongs to the Caesalpiniaceae family. It is commonly recognized as sappan wood and Indian redwood. It has traditionally been utilized in Ayurvedic diabetes therapy [25]. Water extracts of C. Sappan wood (CSW) have been widely used to treat diabetic complications in south-east Asia, specifically in Chinese traditional medicine [26]. CSW exhibited inhibitory activities towards LPS-induced NO production in macrophage, cytotoxicity against human pancreatic cancer cells, breast cancer cell, and colon cancer cell lines [27,28,29,30]. It also showed significant anti-inflammatory activity in many studies [31,32,33]. Moreover, a methanolic extract of *C. sappan* showed significant anti-diabetic activity in alloxan-induced diabetes mellitus in rats and alpha-glucosidase inhibitory activity [34,35]. Brazilin, extracted from CSW, increased the synthesis of fructose-2,6-bisphosphate [36]. Fructose-2,6-bisphosphate synthesized from CS stimulated the process of glycolysis by activating phosphofructokinase-1 resulted in reduced blood glucose levels [37]. Furthermore, some important compounds—including brazilin, sappanol, and episappanol [38]; dibenz[b,d]oxocins protosappanin B, C, and isoprotosappanin B [39]; and homoisoflavonoids 3′-deoxysappanol, 3-deoxysappanone B, and 4-O-methylsappanol [40]—were extracted from CSW by using high-performance liquid chromatography. Saponin, another compound from CSW, exposed potential anti-diabetic activity by inhibiting glucose transport and stimulating insulin secretion in pancreatic β cells [41,42]. CSW also contains tannins capable of lowering blood glucose by inhibiting α-amylase and α-glucosidase, leading to carbohydrate digestion and glucose absorption during high blood glucose levels after meals [43]. Hence, due to the promising anti-diabetic activity of *C. sappan* wood (CSW), we intend to predict its interaction mechanism with metabolic pathways and genes that express proteins involved in the progression of T2DM. 

**Scheme 1:**
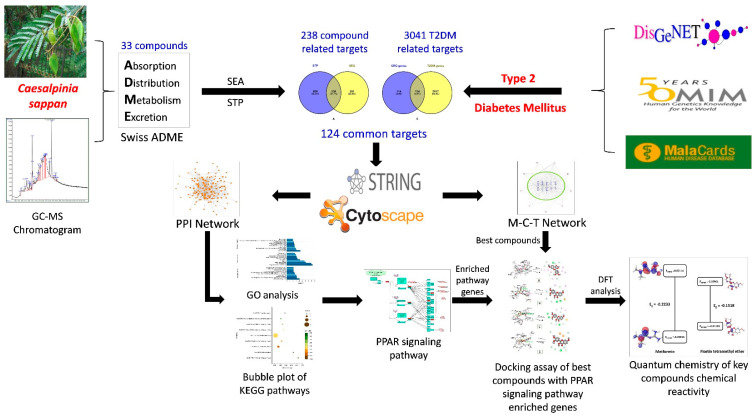
The current framework implies how a comprehensive network pharmacology protocol was used to uncover key targets, possible functions, and pharmacological pathways in T2DM patients treated with MECSW.

A more efficient and safer treatment for T2DM demands a proper explanation of its molecular etiology. Network pharmacology is a well-defined method to investigate the interactions between target compounds, genes, and proteins associated with the disease [44,45]. It can reveal the molecular mechanism of the compounds from a multifunctional point of view, highlighting the interaction of diverse factors involved in the disease progression and elucidating the synergistic impact of the bioactive compounds in a living cell [43,46]. With the advent of modern bioinformatics and systems biology, poly-pharmacological methods contribute suggestively to network-based drug discovery as a cost-effective drug developing tool [47]. Network pharmacology has proven beneficial in explaining the underlying complex relationship between the pharmacological properties of given compounds and the whole biochemical pathway involved in a particular disease [48,49]. In our study, network pharmacology is employed to evaluate the bioactive compounds from CSW and their mechanism against T2DM. Bioactive compounds from CSW are identified using GC-MS analysis. Following that, overlapping genes related to the selected compounds and T2DM are identified using the public databases. Then genes involved in hub signaling are chosen by analyzing gene set analysis. Finally, the most potent candidates of CSW against T2DM are determined by implementing molecular docking analysis for the selected genes. The detailed process is depicted in Scheme 1.

## 2. Materials and methods

### 2.1. Plant Collection and Extraction

*Caesalpinia sappan* wood (CSW) was collected from Chuncheon local market and was authenticated by Dr. Dong Ha Cho, plant biologist and Professor, Department of Bio-Health Convergence, College of Biomedical Science, Kangwon National University. A voucher number (CRT 215) was stored at Kenaf Corporation in the Department of Bio-Health Convergence, and the collected material can be used only for research purposes. The collected dried wood (500 g) was grinded into a coarse powder using an automated grinder. The fined powder (100 g) was soaked in 500 mL of methanol (Daejung, Siheung City, Korea). The mixture was put in a sealed bottle and placed for continuous shaking and stirring (for 3 days) through an automated shaker machine (repeated 3 times for the highest yield). The mixture was filtered (Whatman qualitative filter paper Grade 1) and evaporated using a vacuum evaporator. The evaporated sample (MECSW) was dried under a hot water bath (IKA, Staufen city, Germany) at 40 °C. The yield was found 3.4 g, which was preserved in a refrigerator (−4 °C) for further GC-MS analysis.

### 2.2. GC-MS Analysis

In order to perform the GC-MS study on MECSW, we adopted the GC-MS equipment together with an analytical capillary column DB-5MS (30 m × 0.25 µm × 0.25 mm). Our prior research outlined the whole methodology in precise detail [50].

### 2.3. Filtration of Bioactive Constituents from MECSW

The drug-likeness approach (Lipinski’s rule of five) was used to screen MECSW’s bioactive compounds (identified by GC-MS), which overcome ADME (Absorption, Distribution, metabolism and Excretion) restrictions while securing oral bioavailability score > 0.50. An online program called Swiss ADME [51] was used to explore the drug-likeness properties of identified compounds. In order to do this calculation, we utilized compound’s SMILES from PubChem (https://pubchem.ncbi.nlm.nih.gov/, accessed on 27 July 2021) database.

### 2.4. Acquisition of Compound and T2DM Associated Targets

By using the Homo sapiens mode, we collected targets linked to the filtered bioactive compounds by putting their SMILES into the SEA (Similarity Ensemble Approach) (http://sea.bkslab.org/, accessed on 2 August 2021) and STP (Swiss Target Prediction) (http://www.swisstargetprediction.ch/, accessed on 3 August 2021) databases, respectively. The DisGeNeT (https://www.disgenet.org/search, accessed on 5 August 2021) [52], Malacards (https://www.malacards.org/, accessed on 6 August 2021) [53], and the OMIM (https://www.ncbi.nlm.nih.gov/omim, accessed on 6 August 2021) [54] databases were used to collect targets interacted with T2DM. On the other hand, VENNY 2.1 (https://bioinfogp.cnb.csic.es/tools/venny/, accessed on 15 August 2021) identified and exhibited the common overlapping targets between MECSW bioactive compounds and T2DM targets.

### 2.5. Creating a Network Involving Intersecting Targets

Homo sapiens with a confidence level of >0.4 in protein interactions, the intersected targets between the compound-related gene and T2DM target genes were included into the STRING database Version 11.0b (https://string-db.org/, accessed on 24 August 2021) for protein queries. The Cytoscape 3.8.2 software program [55] was used to subsequently classify the entire network employing the CytoHubba module contained in Cytoscape and following the degree algorithm to identify its key essential genes:

Deg(v) = |N(v)|(1)

where, a node’s neighbors are represented by N(v), while each node’s neighbors are represented by v.

### 2.6. Network Layout for the Pathway Compound Target (PCT)

A graphical depiction of the pathway compound target (PCT) network was created using the preprocessing output of bioactive chemicals from MECSW and frequent T2DM targets that intersected with the MECSW. This network was built using Cytoscape’s merger algorithm plugin (Cytoscape 3.8.2). An analysis of network topology parameters was performed with the help of the network analyzer. Nodes represented bioactive compounds, targets, and pathways, and interactions between these components were shown along the edges. The degree also referred to the prevalence of a node’s interconnected neighbors. The greater the percentage of a node’s that are directly linked to each one, the greater the impact [56].

### 2.7. Investigation of the Role of GO and KEGG Pathways in Common Intersected Targets

The KEGG pathway interpretation and molecular functional annotation of all intersected targets were undertaken on the DAVID (https://david.ncifcrf.gov/tools.jsp, accessed on 19 September 2021) (Database for Annotation, Visualization and Integrated Discovery) database to determine their role in signaling pathways. For this enrichment study, the identifier was OFFICIAL GENE SYMBOL, and the species was Homo sapiens. This database is exceptionally crucial in network pharmacology because it shows targets implicated in disease underlying molecular mechanisms, and the GO database exhibits the descriptive biological terms of those targets, such as biological process (BP), cellular component (CC), and molecular function (MF) [57]. A *p*-value <0.05 was used as a cutoff for GO and pathway enrichment. The *p*-value was corrected using a false discovery rate (FDR) error control technique, and the result was known as the Q value. It was possible to visualize the KEGG pathway bubble plot map visually by using Origin Pro 2021 to examine the routes.

### 2.8. Formulation and Purification of the Ligand and Receptor Protein

The PubChem chemical library was used to obtain the .sdf files of the preferred ligands revealed via compound-target network research along with conventional pharmaceuticals, such as metformin, and co-crystallized protein ligands. As previously reported, the constituents were indeed prepared for molecular docking investigations incorporating the LigPrep tool in Schrödinger Suite-Maestro v12.5 [58]. PPARG (PDB ID: 3E00), PPARA (PDB ID: 1K7L), PPARD (PDB ID: 5U3Q), FABP3 (PDB ID: 5HZ9), FABP4 (PDB ID: 3P6D), MMP1 (PDB ID: 1SU3) and NR1H3 (PDB ID:1UHL), seven receptor proteins essential in hub signaling pathways, were chosen because their crystal structures were available in the RCSB Protein Data Bank (https://www.rcsb.org/, accessed on 11 October 2021) and UniProt database (https://www.uniprot.org/, accessed on 11 October 2021), respectively. Following our previously reported processes, we set the Schrödinger Suite-Maestro v1. 5 integrated Protein Preparation Wizard tools after the 3D crystal structure was located in the RCSB database [59,60].

### 2.9. Glide Directed Molecular Docking Assay

In order to hatch receptor grids overactive molecules (co-crystallized ligand site), we used Glide tools integrated in Schrödinger Suite-Maestro version 12.5 software [61]. Their default topological settings used during grid creation were a scaling factor 1.00, the OPLS3 force field, and a cut-off value of 0.25% for three-dimensional protein assemblies. On the macromolecules’ kernel active site residues, a cubic box of precise facets was placed in 14 Å × 14 Å × 14 Å grid points, at a feasible docking site. Later, docking studies used Glide’s standard precision (SP) scoring system, with each ligand’s best rating conformation and binding result recorded separately.

### 2.10. Quantum Chemistry of Key Ingredients

Utilizing the Jaguar panel of Maestro 12.5 software, the key compound (fisetin tetramethyl ether) and standard drugs’ (metformin) structural variables were absolutely optimized using the Lee–Yang–Parr (B3LYP-D3) correlation functional technique at the 6–31G++ (d,p) level basis set [62]. This optimized configuration also generated enthalpy, Gibbs free energy, highest occupied molecular orbital (HOMO) and lowest occupied molecular orbital (LUMO) border molecular orbital energies using the same level of theory. LUMO energy was deducted from the relevant HOMO energy value to compute each chemical’s HOMO–LUMO gaps (Eg). The following equations estimated the hardness (η) and softness (S) based on border HOMO and LUMO energies.

η = (HOMO − LUMO)/2(2)

S = 1/η(3)

## 3. Results

### 3.1. Exploration of MECSW Ingredients Using GC-MS

**Figure 1:**
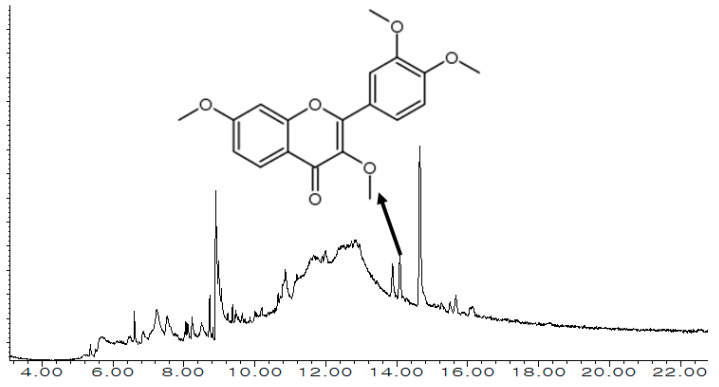
GC-MS peak of the methanolic extract of *C. sappan* and an indication of the key bioactive (fisetin tetramethyl ether).

The data obtained from the gas chromatography-mass spectrometry (GC-MS) study revealed 33 significant bioactive constituents in the MECSW (Figure 1). Table 1 shows the retention time (RT), peak area (percent), chemical formula, and tentative identities of the bioactive compounds. All compounds were categorized (belong to the organic kingdom): namely lipids and lipid-like molecules, benzenoids, organic acids and derivatives, organic oxygen compounds, organic 1,3-dipolar compounds, organoheterocyclic compounds, phenylpropanoids and polyketides, and alkaloids and derivatives.

### 3.2. Drug Candidates Filtering

The primary bioactive ingredients from MECSW were screened based on the drug-likeness properties of recorded small molecules. Those properties were stated as molecular weight must not be over 500, a number of H-bond donor definitely below 10, contrarily H-bond acceptor must not exceed 5, moriguchi octanol–water partition coefficient value must be below or equal to 4.15, and ‘Abott Bioavailability Score’ should be under 0.1 standard value. Remarkably, all of the identified small molecules (33) occupied the above-mentioned criteria and were classified as significant bioactive substances without infringing more than one of the features mentioned earlier (Table 2).

### 3.3. Common Intersected Targets of Compounds within SEA and STP Database

**Figure 2:**
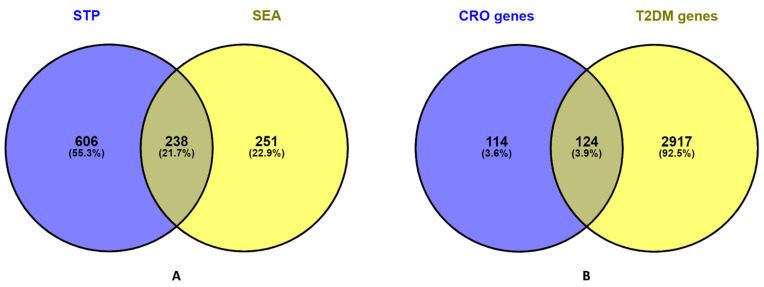
Overlapping genes: (**A**) compounds related genes from SEA and STP; (**B**) T2DM targets from DisGeNet, Malacards, and OMIM.

The screened bioactive compounds were subjected to acquiring compound pertaining targets from public databases. In parallel, each component’s SMILES code was acquired from the PubChem chemical library and entered into the SEA and STP database queries. The removal of duplication targets conveyed the presence of 844 compounds linked to targets from the STP database and 489 targets from the SEA database (Table S1). The result of the Venn diagram analysis disclosed the presence of 21.7% (238) prevalent targets between those 2 databases (Figure 2A).

### 3.4. Potential Overlapping Targets between T2DM Targets and Compound Linked 238 Intersecting Targets

T2DM targets were gleaned from accessing three disease-related public databases, including DisGeNeT, OMIM and Malacards, which resulted in the procurement of 3081 disease-related targets (Table S2). After that, the culled targets were examined with 238 compound-related targets to determine the T2DM targets that were directly connected with the various MECSW compounds (Table S3). Consequently, 124 common targets (Table S4) directly related to T2DM and MECSW compounds were outlined (Figure 2B), in which 26 molecules were closely connected to typical T2DM targets, while the remaining seven compounds, including (3E)-5-Hydroxy-2-methyl-3-hexenoic acid, Disalicylalpropylenediimine, 2-[(4E)-4-Hexenyl]-6-nitrocyclohexanone dimethylhydrazone, 2-(4-methylphenyl) Indolizine, Aspidodispermine, O-methyl-, Nadolol di-methylboronic acid, and 1,3-Dimethyl-5,6-dicarbethoxy-5,6,7,8-tetrahydro-6,7-diazalumazine, were not affiliated with any targets in neither database (SEA and STP). Notably, those compounds were associated with the genes involved in T2DM progression. These compounds were resulting from the intersection of T2DM related genes and target genes related to MECSW compounds

### 3.5. PPI Network Analysis of 124 Common Targets

**Figure 3:**
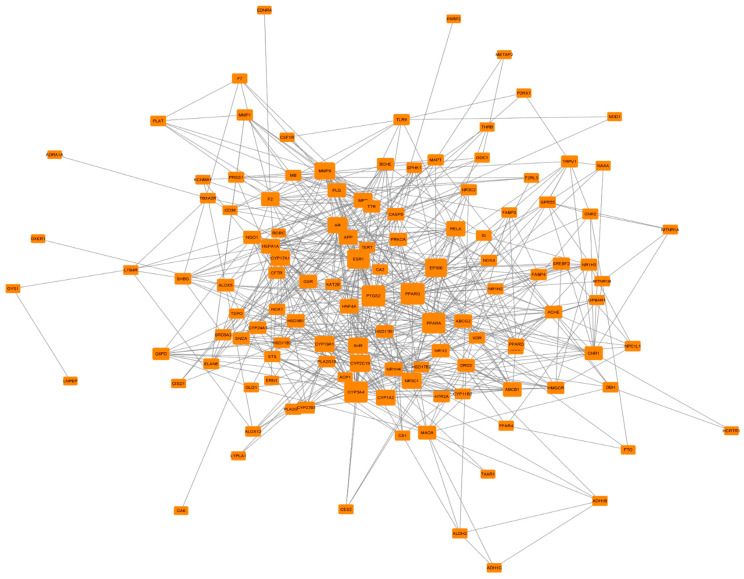
Interactions between proteins (PPI) of common interacting genes utilizing the degree algorithm.

To unearth the potential mechanistic insight of MECSW to treat T2DM, we introduced those 124 common targets into the STRING database to build up a network within them. Meanwhile, the STRING algorithm expressed those 121 nodes connected by forming 596 edges (Figure 3). The average number of neighbors was six, whilst the network diameter was three. However, three targets, namely NEK6, ST6GAL1 and PDE4D, did not interact with any other nodes and were thus omitted from this analysis. To scrutinize the essential key target in the network of T2DM, we further visualized this network in Cytoscape, where we used a degree value algorithm by employing cytoHubba apps. The number of edges connecting to the corresponding target nodes was defined as the number of degrees for each target. Notably, a higher degree value pinpoints the best target in the network. With such conformity, PPARG was designated as a key target (36-degree value) in the network for T2DM progression (Table S5).

### 3.6. Pathway Compound Target (PCT) Network Analysis

**Figure 4:**
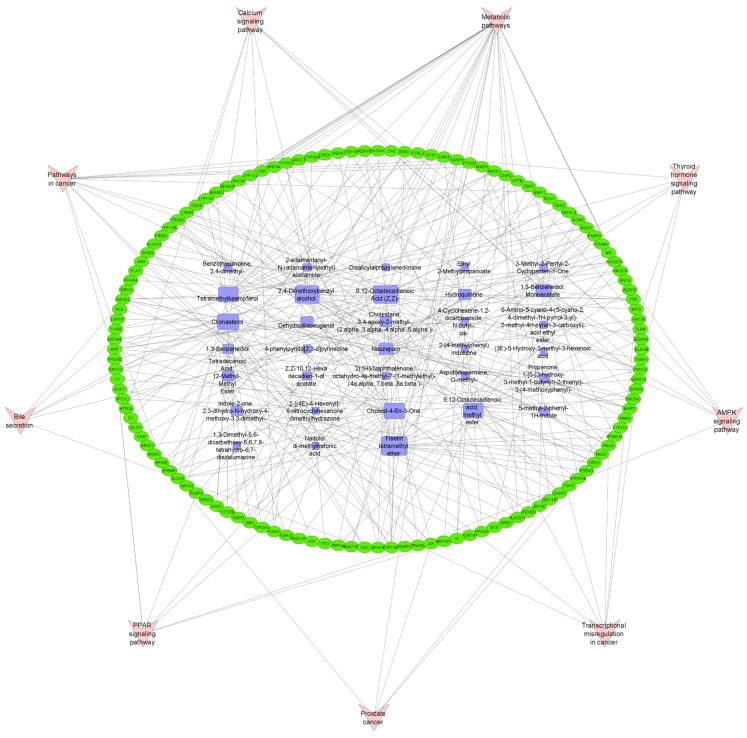
Interactions between MECSW components and common T2DM targets along with their pathways through the pathway compound target (PCT) network.

To ascertain the most significant key components among the detected ingredients from MECSW, we constructed a pathway compound target network utilizing Cytoscape. This network displayed how pathway, compound and T2DM targets correlated among them. The topological parameter analysis of PCT network via network analyzer apps integrated into Cytoscape revealed that this network consists of a total of 166 (121 nodes from common T2DM targets, 33 nodes from MECSW compounds and 9 nodes of KEGG pathway) nodes along with interacting within them through 319 edges (Figure 4), whereas we classified the constituents in the network based on their degree value, referred to as their number of connections to relevant targets. Based on this, the fisetin tetramethyl ether component was picked as a key hub substance (27 degree) in the network that might have an influential therapeutic impact on T2DM (Table S6).

### 3.7. Gene-Ontology (GO) and KEGG Pathway Enrichment Analysis of 124 Common Targets

**Figure 5:**
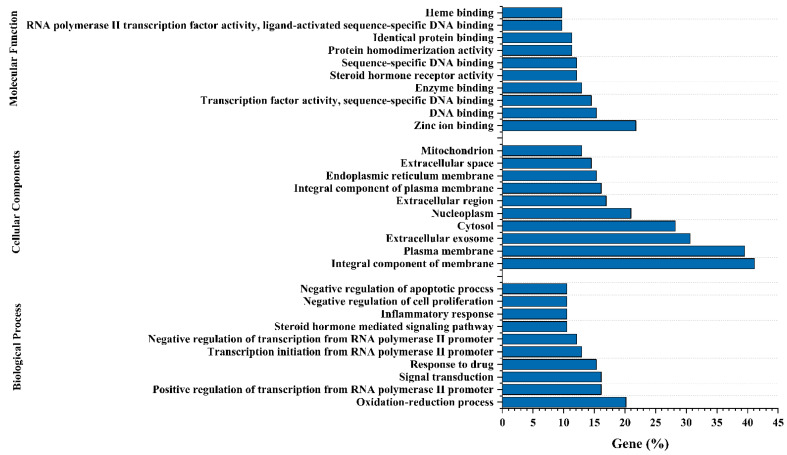
Gene ontology (GO) exploration of compounds’ and T2DM’s shared targets.

The GO and KEGG pathway appraisal truly reflects the intersected 124 common targets engaged in the T2DM functional process through relevant targets molecular function (MF), the biological process (BP) in which it takes part, and its cellular localization (CL). We utilized a web-based tool, “DAVID”, to analyze GO and KEGG pathways. With respect to GO, we determined the top 10 MF, BP, and chemical contents based on the proportion of targets that were enriched in those categories (Figure 5), where MF is mostly involved in binding heme, DNA, ligand activation and sequence-specific DNA, enzyme, protein, zinc ion and protein, and steroid hormone receptor activity. The BP in which those targets significantly participate were apoptotic process, cell proliferation, inflammatory response, positive–negative regulation of transcription, transcription initiation from RNA polymerase II promoter, drug response, and oxidation–reduction process. However, the above activities took place in the following top 10 CLs: mitochondrion, extracellular space, endoplasmic reticulum membrane, an integral component of the plasma membrane, extracellular region, nucleoplasm, cytosol, extracellular exosome, plasma membrane and integral component of membrane.

**Figure 6:**
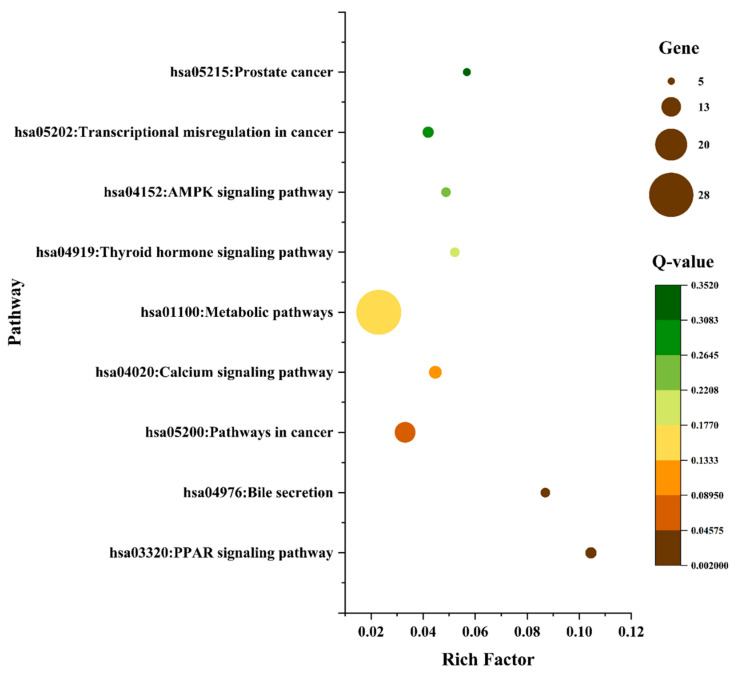
KEGG assessment bubble map showing strongly enriched pathways for common targets.

Similarly, the KEGG pathway enrichment analysis was conducted using DAVID online tool on the 124 putative therapeutic targets for T2DM and discovered 9 KEGG pathways at a threshold level of *p*-value < 0.05. Further, we took into account the rich factor and adjusted the FDR value (Q-value) to define the pathway enrichment analysis, in which the rich factor unveils the amount of pathway enrichment with a substantially lower Q value (Table S7). According to preceding filtering, the PPAR signaling pathway was the most abundantly enriched within the assigned targets, as shown in Figure 6.

### 3.8. Docking Interaction of a Key Substance with PPAR Signaling Pathway Enriched Targets

**Figure 7:**
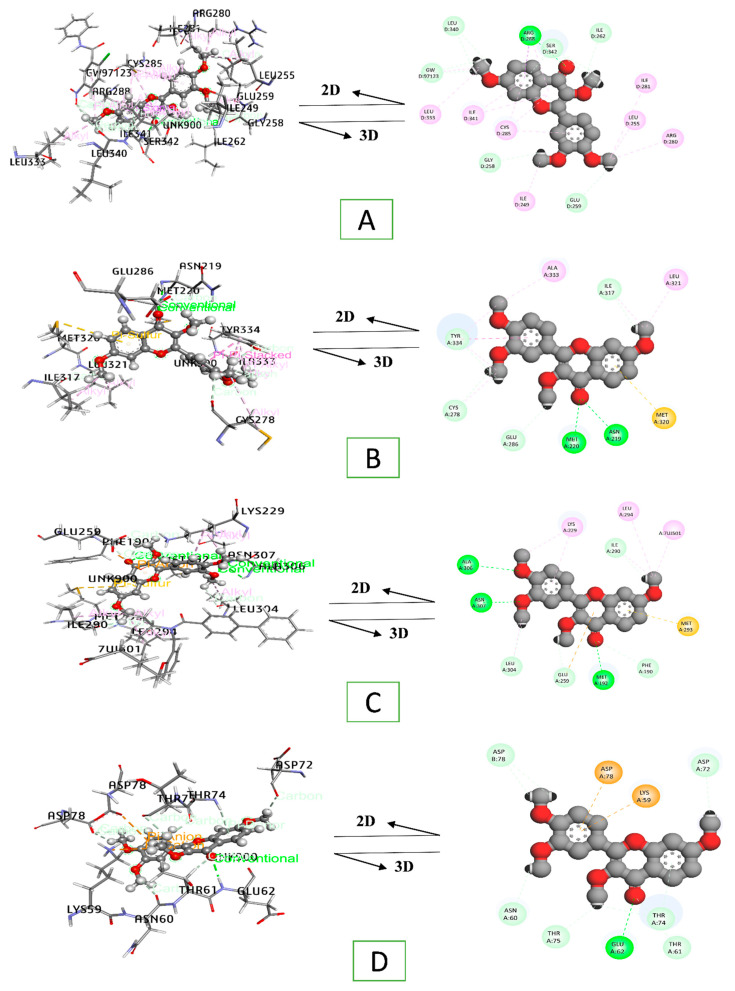

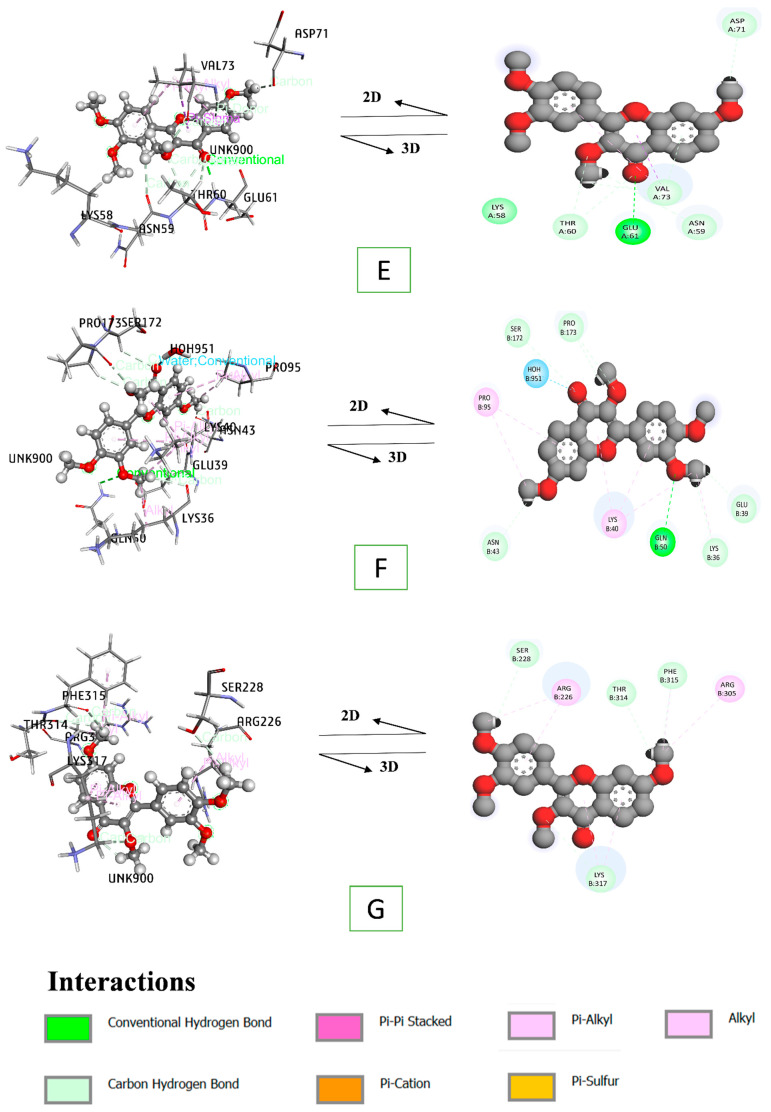
PPAR signaling pathway genes and MECSW key molecules binding interaction. (**A**) 3E00—fisetin tetramethyl ether, (**B**) 1K7L—fisetin tetramethyl ether, (**C**) 5U3Q—fisetin tetramethyl ether, (**D**) 5HZ9—fisetin tetramethyl ether, (**E**) 3P6D—fisetin tetramethyl ether, (**F**) 1SU3—fisetin tetramethyl ether, and (**G**) 1UHL—fisetin tetramethyl ether.

Seven targets of the PPAR signaling pathway involved in the T2DM process were subjected to bind with the selected key compound “fisetin tetramethyl ether” using the Glide tools of Schrodinger. We re-docked each co-crystallized ligand to compare each corresponding complex with their synthesized complex pattern. For additional comparison, we also docked metformin with target transcribed protein molecules. Remarkably, we docked thiazolidine derivative and fibrate medicines as of PPARs agonist to determine their activation energy, whereas PPARG (PDB ID: 3EOO) demonstrated a greater binding affinity of −6.092 Kcal/mol than thiazolidine derivatives (−4.849 Kcal/mol), co-crystallized ligand (−4.381 Kcal/mol) and metformin (−3.761 Kcal/mol) via interacting eleven H-bond of ARG-288, SER-342, ILE-262, GLY-258, LEU-340, and GLU-259 residues; and twelve H-phobic bonds of ARG-288, ILE-249, LEU-333, LEU-255, ARG-280, ILE-281, CYS-285, and ILE-341 residues with fisetin tetramethyl ether (Figure 7A).

The docking feature of PPARA (PDB ID: 1K7L) also reflected the advancement stabilization of 1K7L—fisetin tetramethyl ether interface complex having −5.563 Kcal/mol of seven hydrogen bonds (ASN-219, GLU-286, TYR-334, CYS-278, ILE-317, and MET-220); seven hydrophobic interactions (TYR-334, ALA-333, CYS-278, ILE-317, and LEU-321), and one pi-sulfur bond (MET-320) in contrast to co-crystallized ligand (−5.279 Kcal/mol), fibrate drug (−5.204 Kcal/mol), rosiglitazone (−4.156 Kcal/mol) and the standard medicine metformin (−3.145 Kcal/mol) (Figure 7B).

In the same way, PPARD (PDB ID: 5U3Q) had the most apparent binding affinity towards fisetin tetramethyl ether aiming at −5.58 Kcal/mol, which was markedly different from rosiglitazone (−3.372 Kcal/mol), metformin (−3.319 Kcal/mol) and co-crystallized ligand (−2.845 Kcal/mol) molecules binding energy. Of that prominent interaction was worked out forming eight hydrogen bonds of MET-192, ALA-306, ASN-307, PHE-190, GLU-259, LEU-304, and ILE-290; five hydrophobic bonding of LEU-304, ILE-290, LEU-294, and LYS-229; one electrostatic bond of GLU-259; and one pi-sulfur bond of MET-293 residues (Figure 7C).

Following that, fisetin tetramethyl ether formed nine hydrogens and two electrostatic bonds with GLU-62, THR-61, THR-74, THR-75, ASN-60, ASP-72, ASP-78, LYS-59 and ASP-78 residues of 5HZ9, which all have a significant influence on the FABP3 association (PDB ID: 5HZ9) (Figure 7D). The essence of this interaction complex was −3.924 kcal/mol, opposed to rosiglitazone of −3.339 Kcal/mol, co-crystallized ligand of −3.329 Kcal/mol and a standard drug of −2.908 kcal/mol (metformin).

Fisetin tetramethyl ether binds to the FABP4 (PDB ID: 3P6D) receptor, resulting in −3.816 kcal/mol across nine H-bonds (GLU-61, THR-60, ASN-59, VAL-73, and ASP-71) and three H-phobic bonds (VAL-73), which was considerably lower than co-crystallized ligand −3.74 Kcal/mol, rosiglitazone −3.196 Kcal/mol and the conventional medicine metformin −2.81 kcal/mol (Figure 7E).

The active pocket of MMP1 (PDB ID: 1SU3) association with fisetin tetramethyl ether revealed eight H-bonds with HOH-951, GLN-50, SER-172, PRO-173, GLU-39, LYS-36, ASN-43 and six H-phobic bonds with LYS-36, LYS-40, and PRO-95 residues, ultimately exposing a docking score of −4.043 kcal/mol, which was of much lower energy than the competitive agonist rosiglitazone (−3.79 Kcal/mol) and conventional medicine metformin (−2.74 kcal/mol), but nearly identical to the co-crystallized ligand (−4.365 kcal/mol) (Figure 7F). Meanwhile, the NR1H3 (PDB ID: 1UHL)– fisetin tetramethyl ether complex showed more remarkable affinities of −2.967 Kcal/mol than the co-crystallized ligand (−2.22 Kcal/mol), but lower affinity in the case of standard medication metformin (−4.431 Kcal/mol) and rosiglitazone (−4.983 Kcal/mol) (Figure 7G). Table S8 lists the binding pocket residues in detail.

Nevertheless, compared to the corresponding agonist, the co-crystallized ligand and metformin, the vital essential component of MECSW, showed excellent binding affinity, implying that those binding complexes had greater binding stability than their synthetic counterparts. In contrast, this remarkable finding was not lined with the NR1H3 target in terms of metformin and rosiglitazone, which had a comparatively lower binding affinity. Table 3 provides information on the docking score, co-crystallized ligand, thiazolidine derivatives, and fibrate drug nomenclature.

### 3.9. Quantum Chemistry of Key Ingredients

**Figure 8:**
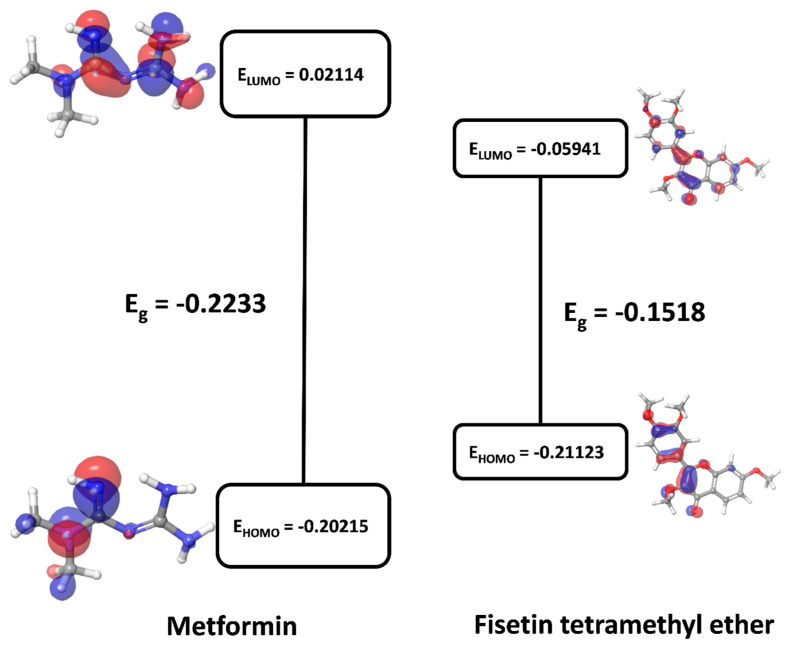
Plotting the energy of key compound and conventional drug on the frontier molecular orbital.

Density functional theory (DFT) was used to figure out molecular descriptors scoring functions, such as enthalpy, Gibbs free energy, HOMO, LUMO, hardness, and softness energy of key compound and standard medicine, to clearly define their reactive chemical nature, structural properties and regions of the molecules. Whilst the negative values of thermodynamic characteristics of fisetin tetramethyl ether are higher than metformin, the thermodynamic attributes of the key compounds are preferably excellent. The compound’s electron-donating and accepting aptitude are symbolized by the HOMO and LUMO frontiers molecular orbitals, respectively. Similarly, fisetin tetramethyl ether also has the highest negative energy of −0.21123 Kcal/mol, signifying that it is the ideal electron donor ingredient than metformin. Hardness and softness are defined as an energy gap, which are determinant hallmarks of compounds’ chemical reactivity [63]. Appropriately, soft molecules have a smaller energy gap over complex molecules, while complicated molecules have an enormous energy difference. Fisetin tetramethyl ether has a relatively low hardness energy of 0.0759 Kcal/mol than metformin. As the responsiveness of medications accelerates with their softness, this prior articulated pattern is equivalent to molecular softness (Table 4). Figure 8 compares the ground state (HOMO) to the first excited state (LUMO) localization pattern of comparable compounds’ frontier molecular orbitals.

## 4. Discussion

A synergistic attributable pattern of multi-pathways, multi-components and multi-targets has constantly been observed in medicinal plants. It is more complex to evaluate the herbal treatment efficiency in one drug-one target model due to the presence of multi-diverse chemical ingredients in plants [64]. To metaphrase the traditional potentiality of medicinal plants, network pharmacology research is an excellent technique that prophesied the therapeutic effects of herbal compounds. Such a strategy unearths their target profiles using a computation-based action plan to generate co-module associations of gene–compound–disease that hypothesize the synergetic rules and network modulatory actions of botanical medicinal formula [65]. In this study, we explored the bioactive present in MECSW employing GC-MS techniques and portended the relevant compound’s target profiles by the sequential intersection of their association with disease targets to dig up the mechanistic molecular process of MECSW in the treatment of T2DM. Pertinently, CSW has been widely used in traditional medicine in the south Asian region. However, the exact mechanism in which it exerts antidiabetic activity has not been clearly elucidated yet. Our current study predicted the mechanistic action of compounds extracted from MECSW at the molecular level. It predicted the interaction of target compounds with the genes and pathways involved in T2DM. Yet, further sophisticated experiments still warrant the establishment of this finding.

Pathway compound target (PCT) network revealed that 124 T2DM genes were linked to 26 chemicals (out of 33 chemicals) and 9 pathways in the diagnostic mechanism of T2DM. Similarly, the KEGG pathway also suggested that the nine pathways (four were signaling pathways) influenced the progression and diagnostic process of T2DM, where the gene ontology (GO) resolution of MF, BP and CC was used to perform the functional process of the common targets between T2DM and compound-related genes. The biological process analysis found that common targets were mainly enriched in oxidation–reduction processes backed by the positive–negative regulation of transcription, transcription initiation, signal transduction, etc., which were possibly located in the integral component of the membrane followed by plasma membrane extracellular exosome, cytosol and others; at a molecular level, the genes participated in zinc ion binding, DNA binding, enzyme binding, and heme binding with notably ligand-activated sequence-specific binding potentiality. The relationship between the nine different types of T2DM related pathways is discussed below.

Prostate cancer: insulin-deprived environment in long-term diabetes results in a lower amount of insulin-like growth factor-1 receptor (IGF-1R), and higher plasma IGF-1 is associated with a higher risk of prostate cancer [66,67,68]. Transcriptional misregulation in cancer: a broad range of human cancer had been correlated with the overexpression of insulin receptors (IR) and IGF-1R [69,70,71,72,73]. Moreover, IR-A activated by IGF-2 promotes metastasis and tumor progression, which is highly correlated with hormone-resistant breast cancer [74]. AMPK signaling pathway: AMPK stimulates glucose uptake and regulates the concentration of AMP in pancreatic beta cells in response to blood glucose levels, suggesting the role of AMPK as a key molecular sensor in regulating glucose and lipid metabolism [75,76,77,78]. Thyroid hormone signaling pathway: the thyroid hormone also regulates glucose homeostasis by increasing hepatic glucose output, altering glucose metabolism, decreasing active insulin secretion, and increasing renal insulin clearance [79]. Hyperthyroidism is associated with poor glycemic control, such as hyperglycemia and insulinopenia, responsible for overt diabetes [80]. Metabolic pathways: T2DM-associated metabolic symptoms resulting from chronic inflammation often lead to cell stress and insulin resistance. Insulin deficiency also interferes with some metabolic pathways resulting in hyperglycemia [81]. Calcium signaling pathway: increased Ca2+ influx was found correlated with increased insulin-mediated glucose uptake, indicating the significance of the calcium signaling pathway in T2DM progression [82]. Furthermore, insulin resistance in humans has been found to be positively correlated with increased serum Ca2+ levels [83,84]. Pathways in cancer: hyperglycemia-induced oxidative stress and DNA damage trigger the primary phases of tumor formation [85,86]. Even high glucose concentrations have been reported to modify the gene expression related to proliferation, migration and cell adhesion, thus leading to cancer [87]. Bile secretion: two of the most prominent signaling molecules in the bile secretion pathway are the farnesoid X receptor (FXR) and the G-protein-coupled membrane receptor (TGR5), which play a significant role in the regulation of lipid, glucose and energy metabolism [88]. The alteration of this pathway may lead to T2DM and relevant metabolic disorders [89]. PPAR signaling pathway: direct action of peroxisome proliferator-activated receptors (PPAR)-γ often results in improved insulin sensitivity, which also acts as major drug target for thiazolidinedione (TZD) in the treatment of diabetes mellitus. TZD activated PPAR-γ inhibits the transcription of genes associated with glucose and lipid metabolism [90,91]. The above mentioned nine pathways are integrally linked to the emergence of T2DM and their rich factors indicate that the PPAR signaling pathway has a higher enrichment level. It was reported that the greater rich factor is particularly linked to the greatest extent of enrichment [92]. Farther, this study only focused on the mechanistic action of one specific metabolic pathway involving the hub gene (PPARG) with highest degree value. Thus, other targets with lower value are not considered in this study. However, they may also possess potential to be a good target, which should be further investigated.

The upregulation of PPARG in the PPAR signaling pathway regulates glucose homeostasis through modulating glucose transporter type 4 (GLUT4), beta-glucokinase, and c-Cbl–associated protein (CAP) in the adipose tissue [93]. In addition, PPARG regulates several adipose-tissue-mediated factors, such as TNF-α, adiponectin, leptin, and resistin, which are essential for insulin sensitivity. Hence, the PPARG agonist is important for increasing glucose tolerance by boosting insulin sensitivity and the functionality of beta cells in diabetics [91]. In contrast, insulin sensitivity may be improved by activating PPARG and PPARA [94]. Severe hyperglycemia has been discerned in individuals with the dominant-negative PPARG genetic defect, suggesting a biological link between type-2 diabetes and the PPARG gene [95]. Furthermore, one of the clock genes, “PPARD”, expressed abnormally to the patients with gestational diabetes mellitus (GDM) and T2DM pregnant women [96]. In that way, activation of clock genes, namely PPARA, PPARD and PPARG, would be a better therapeutic approach for T2DM.

**Figure 9:**
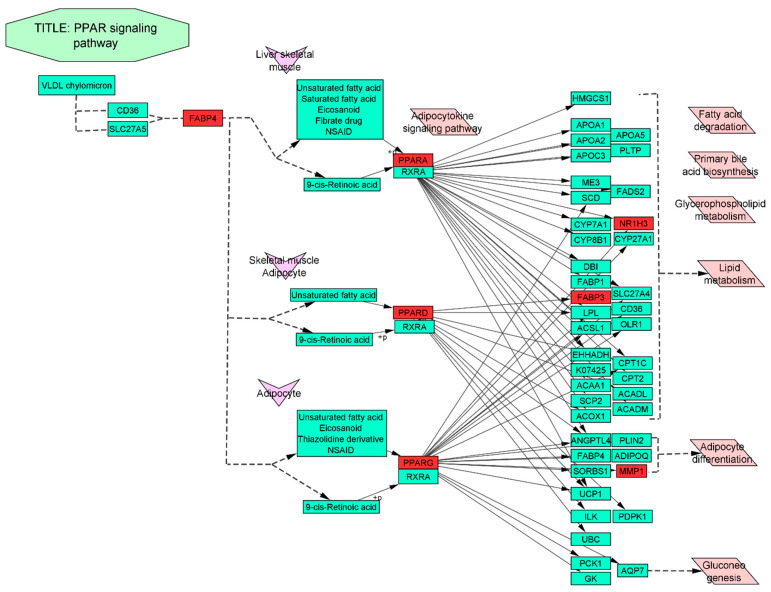
Depiction of the PPAR signaling pathway with the enriched genes in this study are shown by the red node.

In the context of clockwise gene (PPARA, PPARD, and PPARG) activation, we conducted docking assays using the key compound “fisetin tetramethyl ether”, co-crystallized ligand, corresponding gene activation agonist (rosiglitazone and bezfibrate) and the standard medication metformin. As an equivalent to those gene agonists, the key ingredient had excellent activation energy over co-crystallized ligand affinity and metformin binding affinity. This indicates that fisetin tetramethyl ether has the potential to activate PPARs. Several pre-clinical studies also reported fisetin for improved antihyperlipidemic effect and antihyperglycemic effect in streptozotocin-induced diabetic rats [97,98,99]. However, FABP3 and FABP4 also exposed efficient binding affinity and stability with fisetin tetramethyl ether than either metformin or co-crystallized molecules, and MMP1 genes possessed the equivalent or analogous binding interactions and stability positions with respect to the key component and co-crystallized ligand. Compared to the co-crystallized ligand, the NR1H3 gene had more signatory stability, almost equivalent to metformin complex energy. Figure 9 depicts a detailed insight of the PPAR signaling pathway. Due to rosiglitazone’s side effect profile, another contemporary drug, metformin, was used as a control in this study [100]. However, fisetin showed a higher affinity towards PPARG activation than that of both metformin and thiazolidine derivatives.

Additionally, the quantum chemical assessment of the key compound and metformin at the DFT (density functional theory) level was used to verify their chemical reactivity with targets or other species. The HOMO and LUMO energy gaps are essential in influencing medicines’ kinetic stability and chemical reactivity [101]. A large HOMO–LUMO gap can be linked to excellent kinetic stability and inadequate chemical reactivity. Chemical function descriptors, such as hardness and softness, may account for the disparity. Chemical reactivity is exacerbated by their softness [102,103,104]. Simultaneously, our targeted hub component fisetin tetramethyl ether outperformed metformin in robust softness energy. Hence, this progressed as an effective bioactive in treating T2DM. Therefore, fisetin tetramethyl ether might be able to control T2DM. Other studies had also corroborated the antihyperglycemic efficacy of this compound [105,106,107]. However, this research anticipates that fisetin tetramethyl ether could be a multi-target antagonist of T2DM in the network. Even though this research founded the pharmacological effectiveness of MECSW in the treatment of T2DM, more pharmacodynamic and molecular investigations are essential to precisely comprehend the complex synergistic action that underlies the success of MECSW.

## 5. Conclusions

This study hypothesized the molecular insight of the bioactive presence in MECSW to treat T2DM employing network pharmacology. Importantly, this study’s findings show that MECSW might work through targeting the PPAR signaling pathway, where the key bioactive “fisetin tetramethyl ether” exhibited tremendous impact on this pathway in the regulation towards T2DM. These speculative mechanistic actions were further affirmed by molecular docking simulations, in which this key constituent showed excellent binding affinity with each protein agonist involved in the PPAR signaling cascade and conventional pharmaceuticals. In addition, this compound’s quantum chemistry was also characterized by its superior chemical reactivity over the usual medicine. Ultimately, this study provides empirical testimony to substantiate the treatment effectiveness of MECSW on T2DM and outlines profound insights into the bioactive, interacting potential target, and modes of action of MECSW against T2DM, thus adding to the existing knowledge.

## Figures and Tables

**Table 1:** A list of 33 chemical components noted by GC-MS revealed from methanolic extract of *C. sappan* wood (MECSW).

SL. No.	RT Time (min)	Area (%)	PubChem CID	Chemical Formula	Compound Class	Compounds Name
1	5.366	0.22	5363448	C7H12O3	Fatty acyls	(3E)-5-Hydroxy-2-methyl-3-hexenoic acid
2	5.683	1.3	5055	C8H8O3	Phenol esters	1,3-Benzenediol, Monoacetate
3	6.606	0.53	7342	C6H12O2	Carboxylic acids and derivatives	Ethyl 2-Methylpropanoate
4	6.856	0.7	5054	C6H6O2	Phenols	1,3-Benzenediol
5	7.529	2.14	62378	C11H18O	Organooxygen compounds	3-Methyl-2-Pentyl-2-Cyclopenten-1-One
6	8.116	0.52	81750	C9H12O3	Benzene and substituted derivatives	2,4-Dimethoxybenzyl alcohol
7	8.231	0.92	91477	C27H44O	Steroids and steroid derivatives	Cholest-4-En-3-One
8	8.51	1.26	785	C6H6O2	Phenols	Hydroquinone
9	8.731	0.96	5284421	C19H34O2	Fatty acyls	9,12-Octadecadienoic acid, methyl ester
10	8.818	0.15	21206	C16H32O2	Fatty acyls	Tetradecanoic acid, 12-Methyl-, Methyl Ester
11	8.914	3.95	5280450	C18H32O2	Fatty acyls	9,12-Octadecanoic Acid(Z,Z)-
12	8.981	3.34	5363372	C18H32O2	Fatty acyls	Z,Z-10,12-Hexadecadien-1-ol acetate
13	9.375	0.18	41133	C14H24O	Organooxygen compounds	2(1H)-Naphthalenone, octahydro-4a-methyl-7-(1-methylethyl)-, (4a.alpha.,7.beta.,8a.beta.)-
14	10.01	0.22	7210	C17H18N2O2	Phenols	2-((E)-[((E)-2-([(E)-(2-Hydroxyphenyl)Methylidene]Amino)Propyl)Imino]Phenol or, Disalicylalpropylenediimine
15	10.19	0.47	5364132	C14H25N3O2	Allyl-type 1,3-dipolar organic compounds	2-[(4E)-4-Hexenyl]-6-nitrocyclohexanone dimethylhydrazone
16	10.65	0.66	4506	C15H11N3O3	Benzodiazepines	7-Nitro-1,3-dihydro-5-phenyl-2H-1,4-benzodiazepin-2-one or, Neozepam
17	10.73	0.52	610113	C11H13NO3	Indoles and derivatives	Indole-2-one, 2,3-dihydro-N-hydroxy-4-methoxy-3,3-dimethyl-
18	10.88	3.94	610177	C13H9N3	Diazines	Pyrido[2,3-d]pyrimidine, 4-phenyl-
19	11.19	2.93	5116643	C24H37NO	Carboxylic acids and derivatives	Acetamide, 2-(adamantan-1-yl)-N-(1-adamantan-1-ylethyl)- or, 2-adamantanyl-N-(adamantanylethyl)acetamide
20	11.28	1.22	5379033	C20H22O4	2-arylbenzofuran flavonoids	Phenol, 4-[2,3-dihydro-7-methoxy-3-methyl-5-(1-propenyl)-2-benzofuranyl]-2-methoxy- or, Dehydrodiisoeugenol
21	11.65	13.93	5379034	C19H18O3S	Phenol ethers	Propenone, 1-[5-(3-hydroxy-3-methyl-1-butynyl)-2-thienyl]-3-(4-methoxyphenyl)-
22	11.9	1.78	346948	C15H13N	Pyrroles	Indolizine, 2-(4-methylphenyl)-
23	11.99	6.15	610018	C17H18N4O3	Pyrans	6-Amino-5-cyano-4-(5-cyano-2,4-dimethyl-1H-pyrrol-3-yl)-2-methyl-4H-pyran-3-carboxylic acid ethyl ester
24	12.51	11.82	631121	C20H26N2O3	Plumeran-type alkaloids	Aspidodispermine, O-methyl-
25	12.63	3.08	631095	C19H18O6	Flavonoids	4H-1-Benzopyran-4-one, 3,5,7-trimethoxy-2-(4-methoxyphenyl)- or, Tetramethylkaempferol
26	12.73	3.38	631112	C19H29B2NO4	Tetralins	Nadolol di-methylboronic acid
27	12.85	5.34	631171	C19H18O6	Flavonoids	4H-1-Benzopyran-4-one, 2-(3,4-dimethoxyphenyl)-3,7-dimethoxy- or, Fisetin tetramethyl ether
28	12.95	12.81	284060	C12H18N6O6	Diazines	1,3-Dimethyl-5,6-dicarbethoxy-5,6,7,8-tetrahydro-6,7-diazalumazine
29	13.89	1.75	541560	C28H48O	Steroids and steroid derivatives	Cholestane, 3,4-epoxy-2-methyl-, (2.alpha.,3.alpha.,4.alpha.,5.alpha.)-
30	14.09	1.65	91733922	C12H17NO2	Isoindoles and derivatives	4-Cyclohexene-1,2-dicarboximide, N-butyl-, cis-
31	14.65	5.69	457801	C29H50O	Steroids and steroid derivatives	Stigmast-5-en-3-ol, (3beta,24S)- or, Clionasterol
32	15.27	0.4	83247	C15H13N	Indoles and derivatives	1H-Indole, 5-methyl-2-phenyl-
33	15.66	0.68	610182	C15H13N	Quinolines and derivatives	Benzo[h]quinoline, 2,4-dimethyl-

**Table 2:** Documentation of drug-likeness character of 33 compounds from MECSW.

Compounds Name	MW	HBA	HBD	MLogP	Number of Violations	Bioavailability
	<500	<10	≤5	≤4.15	≤1	>0.1
(3E)-5-Hydroxy-2-methyl-3-hexenoic acid	144.17	3	2	0.64	0	0.85
1,3-Benzenediol, Monoacetate	152.15	3	1	1.32	0	0.55
Ethyl 2-Methylpropanoate	116.16	2	0	1.27	0	0.55
1,3-Benzenediol	110.11	2	2	0.79	0	0.55
3-Methyl-2-Pentyl-2-Cyclopenten-1-One	166.26	1	0	2.49	0	0.55
2,4-Dimethoxybenzyl alcohol	168.19	3	1	0.92	0	0.55
Cholest-4-En-3-One	384.64	1	0	6.23	1	0.55
Hydroquinone	110.11	2	2	0.79	0	0.55
9,12-Octadecadienoic acid, methyl ester	294.47	2	0	4.7	1	0.55
Tetradecanoic acid, 12-Methyl-, Methyl Ester	256.42	2	0	4.19	1	0.55
9,12-Octadecanoic Acid (Z,Z)-	280.45	2	1	4.47	1	0.85
Z,Z-10,12-Hexadecadien-1-ol acetate	280.45	2	0	4.47	1	0.55
2(1H)-Naphthalenone, octahydro-4a-methyl-7-(1-methylethyl)-, (4a.alpha.,7.beta.,8a.beta.)-	208.34	1	0	3.41	0	0.55
Disalicylalpropylenediimine	282.34	4	2	1.81	0	0.55
2-[(4E)-4-Hexenyl]-6-nitrocyclohexanone dimethylhydrazone	267.37	3	0	1.82	0	0.55
Neozepam	281.27	4	1	0.9	0	0.55
Indole-2-one, 2,3-dihydro-N-hydroxy-4-methoxy-3,3-dimethyl-	207.23	3	1	1.4	0	0.55
4-phenylpyrido[2,3-d]pyrimidine	207.23	3	0	2.03	0	0.55
2-adamantanyl-N-(adamantanylethyl)acetamide	355.56	1	1	5.2	1	0.55
Dehydrodiisoeugenol	326.39	4	1	2.73	0	0.55
Propenone, 1-[5-(3-hydroxy-3-methyl-1-butynyl)-2-thienyl]-3-(4-methoxyphenyl)-	326.41	3	1	2.43	0	0.55
2-(4-methylphenyl) Indolizine	207.27	0	0	3.32	0	0.55
6-Amino-5-cyano-4-(5-cyano-2,4-dimethyl-1H-pyrrol-3-yl)-2-methyl-4H-pyran-3-carboxylic acid ethyl ester	326.35	5	2	-0.3	0	0.56
Aspidodispermine, O-methyl-	342.43	4	1	2.02	0	0.55
Tetramethylkaempferol	342.34	6	0	0.94	0	0.55
Nadolol di-methylboronic acid	357.06	5	0	1.21	0	0.55
Fisetin tetramethyl ether	342.34	6	0	0.94	0	0.55
1,3-Dimethyl-5,6-dicarbethoxy-5,6,7,8-tetrahydro-6,7-diazalumazine	342.31	7	2	1.22	1	0.55
Cholestane, 3,4-epoxy-2-methyl-, (2.alpha.,3.alpha.,4.alpha.,5.alpha.)-	400.68	1	0	6.68	1	0.55
4-Cyclohexene-1,2-dicarboximide, N-butyl-, cis-	207.27	2	0	1.68	0	0.55
Clionasterol	414.71	1	1	6.73	1	0.55
5-methyl-2-phenyl-1H-Indole	207.27	0	1	3.32	0	0.55
Benzo[h]quinoline, 2,4-dimethyl-	207.27	1	0	3.32	0	0.55

MW = molecular weight: HBA = hydrogen bond acceptor; HBD = hydrogen bond donor.

**Table 3:** Scores for bioactive compound, conventional medication, and their co-crystallized ligands docking with substantially enriched pathway genes of T2DM.

Gene	PDB ID	Compound	Docking Score
*PPARG*	3E00	Fisetin Tetramethyl Ether	−6.092
		Rosiglitazone	−4.73
		Metformin *	−3.761
		2-Chloro-5-Nitro-N-Phenylbenzamide	−4.381
*PPARA*	1K7L	Fisetin Tetramethyl Ether	−5.563
		Bezfibrate	−5.204
		Rosiglitazone	−4.156
		Metformin *	−3.145
		2-(1-Methyl-3-Oxo-3-Phenyl-Propylamino)-3-{4-[2-(5-Methyl-2-Phenyl-Oxazol-4-Yl)-Ethoxy]-Phenyl}-Propionic acid	−5.279
*PPARD*	5U3Q	Fisetin Tetramethyl Ether	−5.58
		Bezfibrate	−4.06
		Rosiglitazone	−3.372
		Metformin *	−3.319
		6-(2-{[([1,1′-Biphenyl]-4-Carbonyl)(propan-2-Yl) amino]methyl}phenoxy)hexanoic acid	−2.845
*FABP3*	5HZ9	Fisetin Tetramethyl Ether	−3.924
		Rosiglitazone	−3.339
		Metformin *	−2.908
		6-Chloranyl-2-Methyl-4-Phenyl-Quinoline-3-Carboxylic acid	−3.329
*FABP4*	3P6D	Fisetin Tetramethyl Ether	−3.816
		Rosiglitazone	−3.196
		Metformin *	−2.81
		3-(4-Methoxy-3-Methylphenyl)propanoic acid	−3.75
*MMP1*	1SU3	Fisetin Tetramethyl Ether	−4.043
		Rosiglitazone	−3.79
		Metformin *	−2.74
		4-(2-Hydroxyethyl)-1-Piperazine ethanesulfonic acid	−4.365
*NR1H3*	1UHL	Fisetin Tetramethyl Ether	−2.967
		Rosiglitazone	−4.983
		Metformin *	−4.431
		N-(2,2,2-Trifluoroethyl)-N-{4-[2,2,2-Trifluoro-1-Hydroxy-1-(Trifluoromethyl) ethyl]phenyl}benzenesulfonamide	−2.22

* = Standard medicine.

**Table 4:** Key chemical and conventional medication quantum properties.

Compound	Enthalpy	Gibbs Free Energy	HOMO	LUMO	Eg	η	S
Fisetin Tetramethyl Ether	−1186.191	−1186.19096	−0.21123	−0.05941	−0.1518	−0.0759	−13.173
Metformin	−432.845	−432.844572	−0.20215	0.02114	−0.2233	−0.1116	−8.957

## Data Availability

All data and materials used are all available in the manuscript.

## Supplementary Materials

The following are available online at https://www.mdpi.com/article/10.3390/life12020277/s1, Table S1: Compound related gene, Table S2: T2DM disease related genes, Table S3: Common genes between SEA and STP, Table S4: Common genes between T2DM related targets and compound related overlapping genes, Table S5: Classification of PPI network ordering based on Degree algorithm for 124 common targets.

## Abbreviations

T2DM	Type-2 diabetes mellitus
PPI	Protein–protein interaction
SEA	Similarity ensemble approach
SMILES	Simplified molecular input line entry system
STP	Swiss target prediction
NR1H3	Nuclear receptor subfamily 1 group H member 3
OMIM	Online mendelian inheritance in man
PPARG	Peroxisome proliferator activated receptor gamma
MECSW	Methanolic extract of *Caesalpinia sappan* wood
FABP3	Fatty acid binding protein 3
PPARA	Peroxisome proliferator activated receptor alpha
FABP4	Fatty acid binding protein 4
PPARD	Peroxisome proliferator activated receptor delta
MMP1	Matrix metallopeptidase 1
KEGG	Kyoto Encyclopedia of Genes and Genomes
FDR	False discovery rate
