# Supplementary material for: Network Pharmacology Study to Reveal the Potentiality of a Methanol Extract of Caesalpinia sappan L. Wood against Type-2 Diabetes Mellitus
Source: Life (Basel). 2022 Feb 13;12(2):277. doi: 10.3390/life12020277 (PMC8880704; doi:10.3390/life12020277)
Supplement: Supplementary file 1 [file life-12-00277-s001.zip › Supplementary Table S1.pdf]

Table S1. Compound related gene:

Genes from STP (844 genes)	Genes from SEA (489 genes)
AKR1B1	RNPEP
PTGDR2	NAALAD2
CPA1	ACHE
CA2	CHRNA3
KMO	CHRNB4
FUCA1	ACR
FBP1	AKR1C3
GPR17	MAOB
SLC13A5	CA12
FOLH1	CA14
CA1	CA5A
CA3	CA5B
CA12	CA6
CA14	CA7
CA5B	CELA1
CA13	BCHE
EGFR	CYP1A1
ACE	CYP1B1
FYN	CTBP2
LCK	HSD17B14
MGAM	HSD17B1
SI	HSD17B2
MME	ELANE
CA9	GABRB1
MAOA	SLC2A4
MAOB	KCNK9
MIF	KYNU
CA7	MMP1
ESR2	MMP9
CA6	MTNR1A
CA4	MTNR1B
CA5A	MB
KDM2A	PDK3
PHF8	PDK4
KDM5C	UQCRB
KDM4E	SRD5A2
GBA	SPR
FUCA2	STS
KDM4A	TTR
SLC16A1	PAM

GABRR1
KDM4C
HCAR2
GLB1
GANAB
KDM3A
KDM6B
FTO
TPMT
CAD
EGLN3
PYGL

ITGAL ICAM1 ITGB2
CASP3
GAA
OGA
UGCG
GABBR2 GABBR1
TDP2
EGLN1
ALPL
NAALAD2
ALOX5
MMP9
MMP1
MMP2
AKR1C3
HSPA1A
AKR1C2
CPA3
CPB2
LTA4H
AMPD3
KDM7A
APEX1
LAP3
REN
AKR1A1
ACHE
PTGS1
PTGS2
BACE1
MAN1B1

METAP2
PIN4
PPID
PPM1B
BRSK2
CA3
CA2
COQ8A
COQ8B
DBH
ESR1
ESR2

CSNK1A1L
LATS1
LATS2
MBD2
OXSR1
PIK3C2G
PIP4K2B
PIP5K1A
PIP5K1C
RIPK4
TRPM6
TYR
GPR84
ALOX5
MPEG1
CDC25A
CDC25C
TYMP
HTR2A
APP
ABCG2
AHR
AKR1C2
AKR1C4
GFER
AOC3
BAZ2A
BAZ2B
BRD7
BRD9
CYP11B1

HSP90AA1
ODC1
NGFR
ADA
MAN2B1
CACNA2D1
CASP6
CASP7
CASP8
CASP2
SLC22A6
F2
ECE1
LIG1
GABBR1
GBA2
ACLY
CYP1A2
MB
ADORA1
ADORA2A
HSD17B3
DAO
NQO2
FADS1
JAK1
JAK2
TYK2
KCNMA1
PNP
GPR84
HDAC6
HDAC2
PARP1
HDAC8
PTPN1
RPS6KA3
GRM5
ALOX15
FEN1
NAT1
COMT
VEGFA
GSK3B
CYP11B2
CALM1
CYP17A1
CYP1A2
CYP2D6
CYP2C19
CYP3A4
CSF1R
DHFR
ERBB3
ERN1
FOS
G6PC
GRIK2
GYS1
JUN
KYAT3
KLK7
RPS6KB2
ABCB1
SLC16A3
NCOA1
NCOA3
NFKB1
NFE2L2
NOX1
NQO1
NQO2
PLA2G2A
PARP10
CD274
CBFB
MPO
PDGFRB
PHLPP2
PPP5C
QPCTL
SLC13A5
SLC5A4
CXCL12
TAAR1
MAPT
TUBB1
RELA

HSD17B2
CDC7
MET
DCTPP1
FABP4

CDK2 CCNA1 CCNA2
CDK9 CCNT1
TTR
PSMB5
IDO1
BRAF
TDO2
PLAU
EPAS1
KDR
ADAMTS5
CXCR2
ERBB2
MKNK1
PDE5A
MGLL
TUBB1
TLR9
NOS1
PLA2G10
CHRNA4 CHRNB2
DHFR
DRD1
SRC
TMIGD3
NOS2
TOP2A
CCNE1 CDK2
DYRK1A
PTPRC
DYRK2
PTK2B
DYRK1B
TNKS2
TNKS
UPP1
KMT5A
CES1

TNFRSF1A
TAS1R1
TAS1R3
TSPO
HSD3B1

ABCB11
ADCY10
AR
SERPINA6
CD4
CDC45
CYP19A1
CRYAB
HSD17B3
HSD17B7
DHCR24
HSD11B2
POLA1
EBP
EPHA1
EPHA2
EPHA3
EPHA4
EPHA5
EPHA6
EPHA8
EPHA7
EPHB1
EPHB2
EPHB3
EPHB6
FGF1
FGF2
G6PD
NR3C1
GPBAR1
NR3C2
CDC25B
ABCC4
NPC1
NPC1L1
NR1H3
NR1H4

SLC9A1
PIM1
GSTA1
PRKD1
PIM2
AURKB
CDK2
CDK1
METAP2
AURKA
MERTK
ELANE
HTR2B
ADRA2A
CDK5R1 CDK5
ADRA2C
ADRA2B
KLKB1
HTR2A
NISCH
PRSS1
CTRC
CTRB1
SLC6A3
CYP19A1
PDE10A
CYP11B1
CYP11B2
CYP2A6
CYP17A1
CTSK
STAT3
HTT
TBXAS1
KCNK2
TYMS
PRKCA
EPHX1
SLC6A4
PDE4A
TAAR1
CHRNA3 CHRNB4
ADRA1A
HRH3
NR1I2
SLC10A2
SLC10A1
ATIC
RORA
SLC22A1
SLC22A3
SRD5A1
SHBG
ST3GAL1
ST6GAL1
SREBF2
VDR
CISD1
QDPR
ABAT
NMUR2
ALDH5A1
ACER2
NDUFAB1
ADH1A
ADH1B
ADH7
APEX1
CERT1
NDUFAF1
CNR1
CNR2
DAGLA
POLB
POLL
POLM
DNM1
ENPP2
CES1
CES2
FAAH
FABP3
GPR174
GPR18
GPR34
HMGCR
EPHX1
EPHX2

CHRM1	KAT2B
LIPE	KAT5
CHRNA4	KDM5A
GRM4	PRKCA
SLC6A2	PRKCE
PSEN2 PSENEN NCSTN APH1A PSEN1 APH1B	LPAR1
CSNK2A1	LPAR2
P2RX7	LPAR3
CHRM5	LPAR4
CHRM2	LPAR5
NOS3	LPAR6
NAAA	LY96
MPO	SELP
GRM2	SLC25A20
GPR35	NAAA
PABPC1	NDUFA1
NR3C1	NDUFA3
PGR	NDUFA2
MCL1	NDUFA5
VDR	NDUFA4
LRRK2	NDUFA7
GABRB3 GABRG2 GABRA5	NDUFA9
PIK3CD PIK3R1	NDUFA8
TGM2	NDUFA6
ADH1A	NDUFA11
CTSH	NDUFA10
CTSC	NDUFA12
CTSF	NDUFA13
CTSS	NDUFB1
CTSV	NDUFB3
ADH1C	NDUFB2
CTSL	NDUFB4
CTSB	NDUFB5
FABP1	NDUFB6
HDAC3	NDUFB7
TRPA1	NDUFB8
PTPN22	NDUFB10
XDH	NDUFB11
MPI	NDUFC1
GABRB3 GABRG2 GABRA1	NDUFB9

GABRA2 GABRB3 GABRG2	NDUFC2
DBH	NDUFAF2
HDAC1	NDUFAF3
RNASEH1	NDUFAF4
TNNC1 TNNT2 TNNI3	NDUFS2
BCL2L1	NDUFS3
SNCA	NDUFS4
TYR	NDUFS5
PDGFRB	NDUFS6
FGFR1	NDUFS1
NQO1	NDUFS7
SQLE	NDUFS8
SRD5A1	NDUFV1
SRD5A2	NDUFV2
AR	NDUFV3
SERPINA6	NOD1
NR3C2	SMPD2
SHBG	MT-ND1
DRD2	MT-ND2
DRD4	MT-ND3
SIGMAR1	MT-ND4L
HSD11B1	MT-ND4
NR1I3	MT-ND5
HTR7	MT-ND6
TRPV1	NDUFA4L2
FAAH	OXER1
CCR5	P2RY10
CHRM3	PAFAH1B2
SIRT2	PLA2G4B
PDE7A	PLA2G5
THRB	PLA2G2C
DRD3	PLA2G10
EPHX2	PDCD4
GABRB3 GABRA3 GABRG2	POLH
GABRG2 GABRB3 GABRA6	POLK
NPC1L1	PPARA
DNMT3A	PPARG
CACNA1B	PTPN13
PPARA	SLC22A6
CTSD	SLC22A8

FABP5
PPARD
MTNR1A
MTNR1B
OPRL1
MAPK3
PRKCH
HSD11B2
PTPN11
AKR1B10
SOAT1
POLB
PLA2G1B
ACP1
PIN1
KCNH2
GSR
CHEK1
CHRM4
GLI2
GLI1
PDE4B
ABCG2
PTPN6
TSPO
P35218
Q9Y2D0
BCHE
NR1I2
PTPN2
CDC25A
ADORA3
PTGES
ESR1
FNTA FNTB
FDFT1
RORA
G6PD
CES2
HMGCR
CYP51A1
MDM2
HSD17B7
HTR6
SLCO2A1
SPHK1
SPTLC2
SPTLC1
IARS1
TLR2
TLR4
TOP2A
TRPV1
ADH1C
GSTK1
ENPEP
BHMT
BBOX1
CBS
CYP4F2
FABP4
FFAR4
GABBR1
GABBR2
GABRQ
GABRR1
GNAI1
GNAI3
GNAO1
GSR
GSTA1
GSTM1
KDM2A
KDM4A
KDM4C
KDM7A
ALOX12
LTB4R
MGLL
PTGER2
PTGER3
PTGER4
PTGFR
PTGIR
ACP1
PPARD
RARB
S1PR2

NPY5R
OXTR
KIF11
CNR1
TACR1
SCN9A
KCNA5
ICMT
CRHR1
CYP24A1
CYP27A1
MDM4
CYP2C9
GPR88
MCHR1
TNF
FABP3
OPRK1
KIT
C5AR1
MAPK10
MAPK9
CXCR3
PIM3
TDP1
IKBKB
CYP3A4
ADCY10
BRD4
ERN1
GRIA2
PPARG
FFAR1
CNR2
SCD
NR1H3
OPRD1
HRH4
SLC6A9
GCGR
MAPK14
GCK
QPCT
IMPDH2
S1PR3
S1PR4
SLC6A11
TBXA2R
TBXAS1
THRB
TOP1
ABCC8
C1R
CA11
CASP4
CASP9
CTSV
CACNG8
CCNE1
CFTR
HSPA1A
IDO2
KCNMA1
PASK
PNMT
PGR
PTPRC
SLC27A2
SNCA
TLR9
TSSK2
HTR1A
HTR7
AADAT
ADRA1A
ADRA1B
ANTXR2
CACNA1S
ODC1
HSD11B1
DRD2
DRD3
FGB
KCNB1
IKBKG
NFATC1
HCRTR1
HCRTR2

HTR2C
GPR119
LIMK2
ROCK2
ASAH1
GRIN1 GRIN2B
CALCRL
PFKFB3
ALOX5AP
EPHB4
CSF1R
ABL1
GRIN2B
RARA
YES1
RORC
OPRM1
CD38
LYPLA1
CASR
LYPLA2
BRPF1
CALCRL RAMP1
BCL2
CHRNA7
CYP26A1
TERT
PTPRF
CSNK1D
FABP2
UGT2B7
NR1H4
CPT1A
PTGER4
RARG
RARB
LDLR
HPGD
GNRHR
PLA2G4A
GRK2
PAFAH1B2
ACACB
SLC1A1
PARP2
S100A4
SLC6A12
SLC6A13
TNKS
ADK
ASIC3
FTO
P2RX7
SLC22A2
SLC47A1
EP300
GLO1
IGF2R
STK17B
GPR183
AKR1C1
SLC1A3
LNPEP
DNTT
PPIB
PPIG
ADORA3
ANO1
MAOA
CA13
CA4
CBR1
CYP2C8
CREB1
EDNRB
ELAVL1
ELAVL3
ERAP1
RCE1
HNF4A
IL2
KCND3
RPS6KA3
LRP6
MDH1
ABCC1
NEK6
NOX4

APP	NR4A2
MTTP	F2RL3
APOB	P4HB
IDH1	PTGS2
CHEK2	ALPI
CDK4	PTPRS
PTGFR	TERT
PLK1	XDH
CLK4	ALDH2
CLK1	AXIN2
CLK2	CTDSP1
GPBAR1	PDF
SMO	EDNRA
LTB4R	GPR55
TOP1	KCNA3
CDC25B	LYPLA1
PDE4D	LYPLA2
ALOX12	PDE4B
PTGER2	PDE4D
SLC22A12	ALPL
PTGER1	RAD51
RXRB	SENP7
PTGIR	CASP7
CD81	GBA2
CYP26B1	AGL
OXER1	GAA
FFAR4	PTH1R
PREP	RGS4
TRPM8	RGS8
MAPK1	SI
IL6	CA1
GLUL	CYP24A1
EDNRA	CYP27B1
CMA1	GLI1
CTSG	NR1H2
CCKBR	RORC
ENPP2	SHH
HNF4A	F2
RBP4	GC
PTGDR	ALKBH3
PRKAG1 PRKAB1 PRKAA2	BCL2L10
TAS2R31	BCAT1
RAF1	F7

PORCN
F2R
LSS
ATP12A
FASN
ACACA
STS
PTAFR
MPEG1
MAPKAPK2
NAMPT
HCRTR2
HDAC10
HPGDS
F10
DAGLA
PLG
PLAT
DGAT1
ALDH2
RASGRP3
MMP13
GLS
PIK3CA
DCK
UNG
AKT1
RPS6KB1
EIF2AK3
CYP27B1
MTOR
MAPT
SSTR4
AKR1C1
GCKR
PGGT1B FNTA
HLCS
PAOX
ADH1B
ADH7
AHR
TBXA2R
PRMT3
DHODH
HPN
ITK
NPY5R
PLG
DHODH
RB1
SMARCA2
VCP
PLAT
PRSS1
PLAU
BDKRB2
CCR8
CD38
CRHR1
COPS5
ENPP3
HPRT1
RAD52
TOP2B

MC4R

GABRA2 GABRB2 GABRG2
SCN2A
SCN10A
HSD17B1
ALDH1A1
CENPE
PPIA
GSK3A
EZR
PDK1
RET

CCNB3 CDK1 CCNB1 CCNB2
PTK2
WEE1
NOX4
NOX1
SLC29A1
MMP3
EP300
PITRM1
SLC5A1
BACE2
THRA
FLT3
EZH2
MAPK8

RBBP4 RBBP7 EED SUZ12 EZH2
MAP2K1
MMP14
PAK4
PBRM1
ANPEP
SMARCA4
ADORA2B
HIF1A
ITGAL
HTR1B
HTR5A
HCRTR1
MGAT2

HTR3A
HTR1A
PDE2A
GABRA1
CDK9
GABRA5
JAK3
SLC6A1
CFTR
FLT1
LDHA
TACR3
MYLK
FKBP1A

GABRA1 GABRB2 GABRG2
PRKCG
CCND1 CDK4
METAP1
RPS6KA5
SIRT1
CASP4
CASP9
PIP4K2C
ADRA1D
IRAK4
CREBBP
PIK3CD
MALT1
ITK
BLM
PDE4C
ROCK1
PRKACA
F2RL1
PIK3C2A
PRKDC
PIK3CB
PIK3CG
PIK3C2B
AADAT
BCAT2
KYAT3
CYP2C19

DPYD
KCNJ5 KCNJ3
TYMP
CXCR1
PHLPP2
DUSP3
RIPK2
HRH2
HRH1
IGF1R
MAP2K7
ADK
ABCC1
ABCC8 KCNJ11
AKT2
S1PR1
ALK
CHUK
RELA
ADAM17
PLA2G6
CEL
CCR4
CCR2
CAMK2D
MAPK13
GLP1R
MAP4K4
ALDH3A1
PDPK1
CSNK2A2
MMP8
GABRB3

GABRB1 GABRG2 GABRA1
CDK1 CCNB1
CDK7 CCNH
DPP7
DPP8
DNM1
ITGA2B ITGB3
NCOR2 HDAC3
DPP9
RNPEP

DNPEP
DPP4
PLA2G2C
ADH4
HDAC11
GRM1
S1PR3
CETP
BRS3
KISS1R
NMBR
NTRK1
NTRK2
NTRK3
SPHK1
SPHK2
CACNA1H
VCP
ABCB1
LNPEP
PLAA
PRKCZ
PLK4
NEK1
AGPAT2
MMP7

CCNE2 CDK2 CCNE1
FPR1
LIPG
MELK
TNK2
GYS1
CACNA2D2
SYK
PDE3A
PDE3B
CDK5
SMYD2
INSR
CCNA2 CDK2
TAB1 MAP3K7
MAP3K7
MAP3K14

CCR1
CCNC CDK8
CDK8
F9
PRCP
MAP3K5
ABCC9
PDGFRA PDGFRB
SGK1
HTR1D
MAP3K11
RAPGEF4
ZAP70
MAP3K9
MAP3K10
AURKC
DLK1
MAP3K2
HPRT1
BMP4
PRKCD
PRKCE
PRKCQ
BAZ2B
BAZ2A
F2RL3
PDGFRA
PDE8B
TNNI3K
PKM
PIK3CA PIK3R1
KCNJ6 KCNJ3
KCNE1 KCNQ1
KCNN4
CBFB
F12
SHH
PDE11A
PDE1A
P2RY1
FAP

CHRNB3 CHRNA6 CHRNB2 CHRNA3
ROCK2 ROCK1

PDE9A
PDE1C
TRPV3
MAP3K12
OGFRL1
PNMT

CHRNA5 CHRNB2 CHRNA4
CHKA
CHRNB4 CHRNA2
CHRNA3 CHRNB2
SLC18A3
MTAP
ATAD2
BLK
CDK6
RPS6KA1
MST1R
FGFR3
CAMK2G
LYN
TEK
WNK2
PLK2
MAPK11
HTR1F
CYP1B1
AXL
NEK2
PTPRS
MPG
GLO1
AKR1C4
DAPK1
AVPR2
ESRRA
MMP12
PKN1
NEK6
NUAK1
ARG1
CAMK2B
CAPN1
HSP90AB1

GHSR
PYGM
SLC27A1
PSENEN
GSTP1
GSTM2
CASP1
KDM4D
PRTN3
SMARCA2
BMP1
CCR3
ST6GAL1
PIK3R1
PLA2G2A
FPR2
DDAH1
ERBB4
LPL
SLC2A1
SERPINE1
ILK
GPR39
AVPR1A
AVPR1B
BDKRB1
POLA1
KARS
BCL2A1
RPS6KA2
TTK
PSMB8
TTL
UTS2R
KCNA3
BRD2
BRD3
PTGER3
HSD3B1
GPR18
CISD1
RGS4
HMOX1
KAT2B

GPR55

XPO1

ECE2

CCND3 CCND1 CDK4 CCND2

SREBF2

NR1H2

DHCR7

GLRA1

PRKCB

DHCR7 EBP

AGTR1

IL6ST

ASIC3

F7

PARP2

CCR8

GRIK2

ESRRB

ST14

NR5A2

NR5A1

TGFBR1

GABRA3

GABRA2

KCNN1

ICAM1

SELE

KCNJ11 ABCC9

NOD1

C1R

PDE6D
