# Supplementary material for: Network Pharmacology Study to Reveal the Potentiality of a Methanol Extract of Caesalpinia sappan L. Wood against Type-2 Diabetes Mellitus
Source: Life (Basel). 2022 Feb 13;12(2):277. doi: 10.3390/life12020277 (PMC8880704; doi:10.3390/life12020277)
Supplement: Supplementary file 1 [file life-12-00277-s001.zip › Supplementary Table S2.pdf]

Table S2: T2DM disease related genes:

T2DM genes within DisGeNeT, OMIM, MALACARDS database (3041 genes)
A1BG
NAT2
ADA
MIR675
CDKN2B-AS1
HOTAIR
ZGLP1
HPN-AS1
APBB1IP
SOD2-OT1
HDAC6
KIF28P
FOXO6
KIR2DS2
BCL2L11
SH2B3
IBD20
INSL5
H3C9P
C4B_2
CDH5
TMED7-TICAM2
AAA1
CHAF1A
PARP2
MIR466
MAMLD1
LINC01672
ARAP1-AS2
OCLN
VIM-AS1
MICA
GJC1
RBM14-RBM4
TMX2-CTNND1

KLRC4-KLRK1
ABCC5
ABCB6
DNM1L
ERVK-20
NR1H3
DNM3OS
IL18BP
PTPRU
ERVK-18
CERT1
ARAP1-AS1
CDH11
PPIF
PGR-AS1
OPN1MW3
HIPK3
CDH13
RASGRP1
LRPPRC
NAMPT
CELA3A
MBNL2
ATP6AP2
WASF2
CDK2
CDK4
LINC01150
EMSLR
TRPM2-AS
LIPC-AS1
TSHZ1
ADAM10
CDK5
NUTF2
TRIM13
DDX39A
GDF11
MRPS31
RABEPK
MIR6835
RIDA
ABCC4

CDK2AP2
CDKN1B

LOC102723407

LOC102724197

LOC102724334
CBSL
STUB1
CDKN1C
SIGMAR1
TP53COR1
CDKN2A
ADAR
CDKN2B
LANCL1
TNIP1
LAMC3
AKR1A1
CDKN3
TFG
KLF2
MICU1
CITED2
SEMA3A
TUBB4B
CORO2B
NOD1
RACK1
PEMT
CPQ
RAPGEF3
NSA2
CD52
RBM14
LYPLA1
C1D
VAV3
TOMM40
FST
TIMM44
CEACAM5
CAP1

CARM1
CEBPA
SEMA4D
CEBPB
FBLN5
CIB2
DEAF1
HYOU1
RNASEH2A
UBD
PERCC1
THRA1/BTR
CERNA3
PRDX4
CECR
AGPAT2
SLC35A1
CEL
SLC19A2
CCT4
CCT2
GNLY
CENPA
SORBS1
SCGN
AHSA1
SLCO1B1
SH2B2
IVNS1ABP
TXNIP
POSTN
IGF2BP2
CST12P
RN7SL263P
KHDRBS1
CELF1
CXCR6
TNFSF13B
CETN1
MTCO2P12
CETP
OGA
NFAT5
TCFL5

MASP2
CTSC
GIPC1
TRAF3IP2
DHS
NEK6
CFTR
SEPTIN9
WASF3
HPSE
PGRMC1
LILRB1
SLC22A7
USP19
MRPS30
PPARGC1A
MALT1
MMP24
CPSF4
ADCY3
UTS2
PAPOLA
SUB1
RALBP1
PRSS21
BTG3
PDIA5
ERP29
EBNA1BP2
KCNQ1OT1
METAP2
IMMT
ERVK-32
SLC27A2
KIF2C
LIAS
CHAT
RAPGEF4
TRIM31

LOC110806263
ESM1
ADAMTS13
NUP42

ADCY5
RPP14
BTN2A1
PTPRT
FAF1
CHGA
CAPN10
SLC7A9
CHI3L1

LOC111674464
PSIP1
WDHD1
CHIT1
TREH
SLC2A6
MAP4K5
IRAK3
AKAP13
POLG2
PRRT2
PTGDR2
DUSP12
IL17F
TREX1
KLF12
LINC02605
SLCO2B1
CHRM3
PARK7
RBPJL
SLC46A1
STK38
CTRC
PHB2
ACOT7
PDAP1
TUSC2
AZIN2
TMEM54

LOC113664106
CHRNA4
ADCY8

DBA2
TXNRD3
UCN3
TNDM1
NLRP3
CYGB
MYSM1
SORCS1
C1QTNF3
C1QTNF5
C1QTNF7
FBXO32
SLC26A9
MARCHF3
GPR146
CISH
H3P8
H3P28
H3P42
H3P10
H3P40
SLC5A11
CKM
ADCYAP1
RMI2
SLC22A12
ERCC8
ABHD15
APOA5
SNAP47
LRG1
ARAP1
TADA1
THEM4
DCD
MRGPRX3
MRGPRX4
RLN3
ADD1
MSS51
CLCN7
CLU
CLK2
SERPINA3

CYP2R1
CLPS
PLIN2
CCR1
DEGS2
CCR5
ACKR2
FOPNL
CMKLR1
NOXO1
LTB4R
CMM
MSI2
ADH1B
ADH1C
OSCAR
NDUFA11
CNP
CNR1
CNR2
CNTF
GJB4
COL1A1
TSACC
COL6A3
TMEM18
RBM45
COL9A3
COL11A2
PLEKHH2
COMP
COMT
COPA
KLF6
CORT
ADM
SLCO6A1
PPARGC1B
ADORA1
GPR151
WDR36
TAAR1
ADORA2A
COX8A

CP
CPA1
ADORA2B
CPB1
CPB2
CPD
KLF14
CPE
CLDN4
CPOX
CPS1
CPT1A
CPT2
CR2
CREB1
ATF2
CREBBP
CREM
CRH
FUNDC1
UPRT
GPR119
CRK
CRMP1
CRP
BPIFA2
CRY1
TRPM6
HT
SIRPA
PARP1
CRYZ
CS
MAPK14
CACUL1
CSE1L
CSF1
CSF1R
PIWIL4
CSF2
CSF2RA
CSF3
CSH1
CSH2

CSK
CSN2
SERPINA12
PWAR1
C2CD4A
NRG4
CST3
WIPF2
CSTA
LYPD4
ADRA1A
CILP2
ZNF569
CTAA1
CTBS
HJV
CTF1
CCN2
CTLA4
PDIK1L
CTNS
CTNNB1
ADRA2A
CTNND1
SIK1
CTRB1
CTSB
CTSD
ADRA2B
PLB1
CTSG
ERFE
CTSK
GPBAR1
RMDN2
CTSL
UPP2
CTSS
CUX1
CX3CR1
CXADR
KLB
PPM1K
ADRB1

CREBRF
CYBA
CYBB
CYC1
ADRB2
SLC2A12
CYP1A1
CYP1A2
AMOT
ADRB3
CYP2B6
CYP2C19
CYP2C9
GRK2
CYP2D7
CYP2D6
CYP2E1
CYP2J2
CYP3A4
LINC00599
CYP3A5
CYP4A11
CYP7A1
CYP8B1
CYP11B2
CBLL2
CYP19A1
CYP21A2
CYP24A1
CYP27A1
SLC35G1
CYP27B1
DAB1
DAB2
CD55
DGKQ
DAPK1
DAPK3
SPRED1
DBH
MARCHF10
SLC16A11
DBP
DCN

ACE
DCX
TMPRSS6
DDIT3
DDOST
OXER1
DDT
TLE5
DECR1
DEFA1
DEFA3
SGMS2
DEFB1
DES
SLC30A8
GLIS3
NLRP6
PTCRA
DHPS
NQO1
DIAPH1
AFM
DIH1
DIO2
DIO3
DKC1
DLAT
DLD
AFP
DLX3
AGA
DMD
SARDH
DMPK
DMRT1
DNAH8
AGER
DNASE1
DNM2
DNMT1
DNMT3A
DOCK3
DPP4
DPT

DPYD
DPYS
AGRP
JAG1
ATN1
DSC3
RCAN1
AGT
DSG3
DSPP
HBEGF
DUSP1
DUSP4
AGTR1
DUSP9
DYRK1A
AGTR2
TOR1A
E2F1
APLNR
EBF1
ECE1
EDA
ABCA1
S1PR1
EDN1
EDNRA
EDNRB
AIC
EEF2
EFNB2
EGF
CELSR2
EGFR
EGR1
AHR
EIF2S1
ARID2
PAOX
EIF2S3
AHSG
UBR1
EIF4E
EIF4EBP1

EIF4EBP2
EIF5A
AIF1
CELA1
ELANE
SERPINB1
ELAVL2
A2M
CRTC2
ELK3
ELN
ZBTB7C
ENG
ENO1
ENO2
ENPEP
PRSS55
EP300
NRK
PDILT
EPHB1
EPHB2
EPO
EPOR
ERBB2
ERBB3
ERBB4
ERCC1
ERCC2
EREG
AKT1
ERG
AKT2
ERN1
FBL
ESD
ESR1
ALAD
ESRRA
ALAS1
ETS1
ETV3
ALB
EXT2

ALCAM
EZH2
F2
F2R
F2RL1
F3
F5
F7
F8
F9
F10
ALDH1A1
F11
FABP4
FABP1
FABP2
ALDH2
FABP3
FABP5
ACSL1
PTK2B
FAP
FBLN1
FASN
FAT1
LRRC55
FOLH1B
TPCN2
FBN1
FBN2
EFEMP1
FBP1
RANP1
PHACTR1
FCGRT
HOXA11-AS
JAZF1
FDPS
GPRC6A
RFX6
GPC4
FES
FGB
FGF1

FGF2
ABCD2
FGF13
FGFR1
FGFR4
FGL1
FHL1
FHL2
MRAS
PUF60
VASH1
AAK1
ANKRD26
NLRP1
INPP5F
FKBP4
FKBP5
CARD8
SACM1L
KLRK1
MMRN1
MSRB2
ATF6
SIRT2
DKK1
TPX2
PDCD11
PALLD
UNC13A
MYO16
FOXC2
FOXM1
FOXO1
FOXO3
AKR1B1
NFASC
FLG
KDM6B
FLII
FLNA
LPIN1
PASK
ATG4B
FLT1

TBC1D1
NBEAL2
FBXO28
PLCB1
PHLPP1
FLT4
KAZN
MCF2L
CLEC16A
FMO3
CUX2
FN1
ZNF629
SIK3
SIRT4
SIRT3
SIRT1
TARDBP
CELA3B
ANGPTL2
ABCB10
FOLH1
NPTXR
TRAM1
BRD4
FOLR1
PES1
FOLR2
ZFYVE26
CABIN1
FOS
NNT
FOSB
RBFOX2
DDAH2
LPAR3
PADI4
DDAH1
SMUG1
ELP5
LEMD3
DAPK2
CD2AP
SH3BP1

BACE1
NUP62
PLA2G15
TMEFF2
RAB38
TMEM245
DIANPH
ALOX12
FXN
ALOX5
ALOX5AP
PANX1
PRPF6
ZNF318
ALOX15
IL27
MTOR
ALPI
FSHMD1A
NR5A2
ALPP
NR5A1
GAST
FUT2
FNDC5
KDSR
G6PC
G6PD
ANGPTL5
SLC37A4
CNIH2
GABPA
PCSK9
PTF1A
TCERG1L
WDR72
FRMD3
GAD1
NEGR1
GAD2
GCA
TAFA5
ARIH1
TPSG1

BACE2
TXN2
PART1
GALNS
POC1A
PAMR1
RNF19A
AMBP
GALNT3
NDUFAF3
MRGPRX1
SAMHD1
TPGS2
KANK2
GAP43
GAPDH
SH2B1
TIPARP
CHMP2B
SNED1
GORASP2
TKFC
ACOT11
COPD
APPL1
POLDIP2
LRIT1
ZBTB20
GAS1
PTPN22
AMD1
GAS6
PHGDH
GATA3
GATA4
FBXO25
FBXO8
GATA6
TINF2
GATM
ANKRD2
FGF21
AMD1P2
GBE1

GAPDHS
GC
GCG
GCGR
GCH1
GCK
GCKR
SLC17A5
OPN1MW
GDF2
BSCL2
GREM1
MSTN
GDF10
HS6ST3
GDNF
GEM
GFAP
GFER
GFPT1
HAVCR1
GGT1
AMH
GH1
GHR
GHRH
GHSR
CBLIF
GIP
GIPR
GJA1
FETUB
ABL2
AMPD1
GJA3
GJA4
GJA5
FOXD3
ATP2C1
NOX1
SIGLEC7
LAT
SND1
GJB3

FOXP1
TAF5L
GK
CACYBP
INTU
NAAA
GLS2
GLA
IL17B
HCAR1
GPR162
PDCD4
GCLC
HPGDS
ANGPTL3
RPS6KA6
DESI1
SGSM3
GLI2
GLO1
GLP1R
MAT2B
TOR2A
GLRX
GLUD2
GLUL
GNAO1
GNAS
GNAT1
GNB3
AMY2A
ABO
GOT2
GP1BA
SLCO1B3
IFNL3
ANG
NEAT1
OR10A4
CXCR3
HECTD4
KSR2
CHAMP1
UTS2R

ANGPT1
CAVIN1
ZNF763
RSPO1
BTBD8
MCHR1
ANGPT2
IGHD1-7
DLL1
GPER1
GPR35
TRBV7-8
NSMCE2
GPR39
FFAR1
GPR42
FFAR2
GPT
GPX1
GPX3
GPX4
GRIA2
REM1
DEXI
LAMTOR2
GRN
OSTM1
FLVCR1
DBNL
ANPEP
GRIN1
GRIN2A
GRIN2B
SETD2
NDUFAF4
NR3C1
STXBP6
CNIH4
SLC25A4
DROSHA
TBK1
GRM2
CD274
GRM5

CXCL1
GSK3B
GSN
GSR
GSTA4
GSTM1
GSTP1
GSTT1
GTF2H1
GTF2H4
GRHL1
TRPM5
ICOS
NPC1L1
GPSM2
GUSB
EEF2K
NME7
SEC61A1
GYPA
GPR132
NENF
GYPB
DSE
PADI1
CERS2
NRBP1
GYPE
PSAT1
GYS1
SH3KBP1
GZMB
H1-2
ANXA1
ANXA2
HABP2
HARS1
HAS2
HBA1
HBB
HBG2
HCLS1
HCRT
HCRTR1

HDAC2
CFH
HFE
ANXA5
ERVW-1
HGF
NT5C
NRG1
PIK3R4
HHEX
ANXA6
HIF1A
UBE2K
HK2
ACACA
HLA-A
HLA-B
HLA-C
HLA-DMA
HLA-DMB
MNX1
HLA-DPB1
HLA-DQA1
HLA-DQA2
HLA-DQB1
HLA-DQB2
HLA-DRB1
AOC2
HMGB1
HMGCR
HMGCS2
HMGA1
HMOX1
NR4A1
FOXA2
HNF4A
HNF4G
ONECUT1
SLC29A2
BPIFA4P
HNRNPK
ACACB
HOXA3
HOXC4

APC
HP
HPN
APCS
AIRE
HPX
MMAB
AGFG1
HRH1
HRC
PRMT1
HES1
HSD3B1
HSD11B1
HSD11B2
HSF1
HSPA1A
HSPA1B
HSPA2
HSPA4
HSPA5
XIAP
HSPA9
HSPB1
HSPB2
HSP90AA1
HSP90AB1
HSPD1
HSPG2
NDST1
HTC2
APOA1
HTR2A
HTR2C
APOA2
APOA4
TNC
IAPP
IARS1
IBD2
APOB
IBSP
ICA1
ICAM1

GPIHBP1
HCAR2
FFAR4
TMEM119
BRINP3
ARMH1
ID1
GADL1
IDDM3
IDDM4
TSPAN33
IDDM7
VWA2
IDDM11
IDE
GOLGA6A
NANOS3
CYCSP51
IFNA1
IFNA13
APOC3
IFNAR1
IFNAR2
IFNG
ZFP57
SOX2-OT
PWAR4
IGF1
APOE
IGF1R
IGF2
IGF2R
IGFALS
IGFBP1
IGFBP2
IGFBP3
IGFBP5
IGFBP7
CCN1
APOH
GPR142
APP
APRT
ZACN

SLCO4C1
TICAM2
KLK3
FAS
IL1A
IL1B
IL1R1
IL1RN
IL2
IL2RA
IL2RB
IL3
IL4
IL4R
IL6
IL6R
IL6ST
IL7
IL7R
CXCL8
CXCR1
IL9
AQP1
IL10
IL12B
IL13
ACADSB
IL15
IL15RA
TNFRSF9
IL17A
IL18
FOXK2
ILF3
AQP4
ILK
IMPDH1
IMPDH2
IDO1
ING1
ING2
CXCL10
INS
INPPL1

AQP7
INSL3
INSM1
INSR
INSRR
PDX1
IRAK1
IRF1
AQP9
IRF7
IRS1
ISG20
AR
ISL1
ITGAM
ITGAX
ITGB2
ITGB7
ITIH1
ITPK1
ITPR1
ITPR3
IVD
JAK1
JAK2
JUN
JUNB
JUND
GSTK1
AREG
NDUFS7
KCNC2
KCNE1
KCNH2
ENHO
KCNJ1
KCNJ3
KCNJ5
KCNJ6
KCNJ9
KCNJ11
KCNMA1
KCNN4
KCNQ1

LINC-PINT
MALAT1
KDR
KIF5B
KIR2DL2
KIR2DL3
KIR3DL1
KISS1
KLK1
KLKB1
KNG1
ARG1
KRAS
KRT5
KRT8
KRT16
SUMO4
TBPL2
KRT18
TMEM189

TMEM189-UBE2V1
SERPINA13P
LINC01550
C2CD4B
CCL4L1
C1QTNF12
LIN28B
KTN1
MAFA
LAD1
C1QL3
HES3
LAMP1
LAMC2
LAMP2
RPSA
LBP
LCK
LCN1
LCN2
LCP1
LDHB
LDLR

LEP
LEPR
LETM1
LGALS1
LGALS2
LGALS3
LGALS3BP
LGALS4
LHX1
LIF
LIG4
LIPC
LIPE
FADS1
SMIM10L2A
LMNA
LINC02694
LNPEP
LOX
LOXL2
LPA
LPL
LRP1
LRP2
LRP6
LRP5
LRPAP1
LINC01194
GTF2H5
LTA
ARNT
LTBR
ARNTL
MIRLET7G
LYZ
MIR107
MIR122
MIR126
MIR130A
MIR132
MIR134
MIR140
MIR142
MIR143

MIR145
MIR146A
MIR148A
MIR149
MIR150
MIR152
MIR155
MIR15A
MIR15B
MIR181C
MIR182
MIR184
MIR185
MIR190A
MIR199A1
MIR199A2
MIR19A
MIR200A
MIR200B
MIR200C
MIR203A
MIR204
MIR205
MIR21
MIR210
MIR211
MIR214
MIR216A
MIR217
ARR3
MIR22
MIR221
MIR222
MIR223
MIR23A
MIR23B
MIR24-1
MIR25
MIR27A
MIR27B
MIR29A
MIR296
MIR29B1
MIR29B2

MIR29C
MIR302A
MIR30A
MIR30D
MIR30E
MIR31
MIR320A
MIR34A
MIR93
MIR96
MIR98
MIR99A
MIR17HG
MXD1
SMAD1
SMAD2
SMAD3
SMAD4
ARRB2
SMAD7
MAFD2
ASIC1
ARSA
STS
MAOA
MAP2
MAP6
MAPT
MAS1
MB
MBL2
MBNL1
MBP
MC2R
MC3R
MC4R
MCL1
SMCP
ADAM11
DNAJB9
MDH2
MDM2
MDM4
MECP2

MEF2A
MEFV
MAP3K5
MEN1
MEOX2
MET
MFAP1
MFGE8
MGAT2
KITLG
MGP
MIF
CXCL9
MIP
MITF
MLH1
MAP3K9
MAP3K10
MLN
ACHE
FOXO4
NR3C2
MME
MMP1
MMP2
MMP3
MMP8
MMP9
MMP10
MMP14
MNAT1
ASIP
MPG
MPI
MPO
MPST
MPZ
MRE11
ABCC1
MS
RNASEK
MIAT
CDNF
VN1R17P

GPR166P
MIR330
MIR338
MSH3
MSMB
MST1
MSX2
MT1A
MT1E
MT1JP
PLF
MT2A
ATP6
TRNC
COX1
COX2
COX3
CYTB
MTHFR
MTM1
ND1
ND2
ND3
ND5
ND6
MTNR1A
MTNR1B
MTTP
TRNF
TRNK
TRNL1
TRNQ
TRNS1
TRNS2
TRNV
TRNW
MUC1
MUSK
MMUT
MUTYH
MVD
MYC
MYD88
MYF6

SERPINC1
MYH9
ZFHX3
MYL2
PPP1R12A
NAB2
NAGLU
ATF3
ATF4
NUBP1
NBN
NCAM1
NCK1
NCL
NDN
NDP
NDUFA1
NDUFA6
NDUFB3
ATIC
NDUFB9
NDUFB10
NDUFS1
ATM
NDUFS2
NDUFS3
NDUFV1
NDUFS4
NDUFS6
NDUFS8
NDUFV2
RERE
NEFL
NEUROD1
NFATC1
NFATC3
NFATC4
NFE2L2
NFIL3
ATP12A
NFKB1
NFKB2
NFKBIA
NGF

NGFR
NHS
NIDDM1
NIDDM2
NINJ1
NNAT
NM
NME1
NNMT
NOS1
NOS2
NOS3
NOTCH1
NPY
NOTCH2
NOTCH3
CCN3
FXYD2
NPHS1
ATP2A1
NPPA
NPPB
ATP2A2
NPPC
SLC11A2
NRF1
ACR
ATP2B1
NRTN
YBX1
ATP2B2
NTS
NTSR1
NUCB2
NUMA1
TRIM72
CISD2
OAS3
OAT
MIR375
MIR377
MIR424
ATP4A
ODC1

ODF1
OGG1
OGN
OLR1
OPA1
TNFRSF11B
ORM1
CLDN11
OXA1L
OXTR
P2RX3
P2RX4
P2RX5
P2RX7
P2RY1
P4HB
PA2G4
FURIN
PAEP
RRM2B
NOX4
PRDX1
SERPINE1
IL20
IL22
MBL3P
PAM
NEUROG3
REG3A
PAPPA
PRKN
PARN
PAWR
PAX2
PAX4
PAX6
ASAP1
AK3
F11R
PBX1
PC
PCBD1
PDE11A
FOXP3

SOST
PCDH8
TPRKB
ASCC1
TMED7
TVP23B
SERPINA5
PCK1
PCK2
GAL
MLXIPL
ADIPOR1
PCMT1
NDUFAF1
TFB1M
METTL9
PCNA
RMDN1
IER3IP1
ANGPTL4
INSIG2
SDF4
PCNT
DCTN4
CYB5R4
HSPA14
GP6
GLRX5
PCSK1
MZB1
NT5C3A
PCSK2
CLEC1B
TLR7
PCYT1A
TIMMDC1
PDB1
PDC
PDCD1
DACT1
PRKAG2
POLK
PDE4D
DCDC2

ISYNA1
HSD17B7
PDE7A
DTL
SIRT7
SIRT6
RAB14
PDGFRA
IL23A
UFM1
GDE1
PIAS4
TRIM33
NBAS
ATP6V1H
SF3B6
RASD1
PDK4
MPC1
ZFR
ENPP1
EMCN
PDR
GHRL
ARID4B
PECAM1
RTEL1
SERPINF1
MAP3K20
ADA2
PER1
PEX1
PEX6
PF4
PF4V1
ACP1
ATP5PF
PGC
PGF
PGM1
ABCB1
PHB
SLC25A3
SERPINA1

SERPINB6
PIK3CA
PIK3CB
PIM1
PIK3CD
PIK3CG
PIK3R1
PIN1
PKD1
PKLR
PKM
PKNOX1
PLA2G1B
PLA2G2A
PLAG1
PLAGL1
PLAT
PLAUR
PLCB3
IL17D
UBASH3A
PLCG1
HDL3
PLG
PLEK
SERPINF2
PLIN1
PLK1
PLN
PLTP
PLXNA1
PLXNA2
PNOC
PMM2
PMP22
IL20RA
SLCO1C1
ACP5
FAM3B
TLR9
H2BS1
PODXL
ERRFI1
TREM2

TREM1
POLD1
POLG
SIAE
POMC
YIPF1
SEMA5B
PON1
PON2
MOV10L1
PON3
POR
KRT20
POU1F1
SMOX
ATR
POU2F1
C20orf181
POU2F2
CCHCR1
ROBO4
NDUFB11
DDIT4
EPB41L4B
DLL4
POU4F1
ARL15
PPARA
PPARD
PPARG
KIF1A
FEV
FBLIM1
PPIA
TET2
CNNM2
DYM
PPID
TRPM7
BCAS3
VPS13C
NCAPG2
CASZ1
CDKAL1

SARS2
ACP3
TUG1
MARCHF1
MOCOS
PPP1R3A
SLC52A1
PPP1R3C
AVP
ANO1
WRAP53
MTPAP
MSTO1
RMDN3
AVPR1A
RCBTB1
PTPA
SLC47A1
RHOT1
FBXW7
AVPR1B
SLC29A3
GIMAP5
CHDH
HEMGN
IMPACT
MEG3
PPY
AVPR2
MIR429
MIR449A
PREP
NOP10
SLC30A10
MAML3
SOX6
PLXNA3
FOXRED1
MIOX
ZCCHC8
RNPC3
ITLN1
CARMIL1
FERMT1

PRKAA1
STAP2
PRKAA2
SYBU
PRKAB1
NHP2
PRKACA
PRKAR1A
ENAH
PRKCA
PRKCB
PRKCD
KDM3A
PAG1
SELENOS
CISD1
USE1
ACOT13
TMEM126B
GSDMB
GPRC5C
ZNF395
ACSS2
ZC4H2
ANGPTL8
LANCL2
APOM
MAPK1
MAPK3
NSFL1C
MAPK7
MAPK8
MYDGF
MAP2K3
MAP2K6
DNAJC3
PCDHGA4
PRL
PSPN
PROC
ZNF253
PROS1
PROX1
TRPV5

PRSS1
PRSS2
MASP1
PRSS8
CYP26B1
SLC2A9
ASAH2
PSEN1
PSEN2
CLDND1
TWNK
SUCNR1
B2M
RETN
SPHK2
PSMA6
NMUR2
ENY2
PYY
FAM20C
CHPT1
ADAMTS9
BAAT
ACKR3
AKR1B10
COQ9
PCBP4
PNPLA2
KMT5AP1
CAMK1D
GOPC
PSMD7
PSMD9
PSMD10
PELI1
ADAMTSL3
BAD
PSPH
PTBP1
PTCH1
PTEN
PTGDS
JPH3
GJD2

PTGIS
MIR433
HNP1
PTH
AS3MT
PTGS1
PTGS2
PTHLH
MIR409
MIR146B
MIR497
PTH1R
MIR499A
MIR503
AHRR
SEMA6A
CFAP97
SHROOM3
CIP2A
RNF213
PTPN1
PTPN2
TRIB3
PTPN6
MARK4
SUGP1
HAMP
G6PC2
ZNF410
PTPRD
PTPRF
PTPRN
PTPRN2
PTX3
BAX
RAD1
NEUROD4
PVR
NECTIN1
BBS1
PVT1
BBS2
PCTP
OPRPN

ARHGAP22
RAC1
RAC2
MOK
RAF1
RAG2
BCHE
RAN
IL21
RAPSN
RARRES2
ACE2
UBL5
GER
TRPV4
SCPEP1
LGR6
NPS
RBP3
CCND1
RBP4
RCN2
OPN1LW
BCL2
REG1A
REG1B
BCL2A1
RELA
REN
RENBP
REST
RFC2
RGS1
ACTB
RHD
RHEB
RHO
RIT2
RNASE2
RNASE3
SLC25A19
BRD2
BACH2
ELOVL5

NIF3L1
IGAN1
GAS5
PROK2
RNY1
RNY3
ROBO1
ROCK1
RORC
ROS1
HCN2
RP9
BCR
RPL10
RPL29
RPL36A
MBOAT4
RPS6
DELYQ11
RPS6KA1
HCC
MIR483
MIR486-1
RPS6KB1
RPS19
RREB1
RXRG
RYR2
RYR3
BDNF
S100A1
SORT1
S100A8
S100A9
S100A10
S100A12
S100B
CFB
ACSM3
SALL1
SATB1
SCD
BGLAP
CLEC11A

SCN2A
BGN
SCN7A
SCN10A
SRL
CCL2
CCL4
CCL5
CCL11
CCL16
CCL20
CCL21
CCL22
BID
CXCL11
CXCL5
SRR
SDC4
CXCL12
THADA
UBE2O
ELMO2
CIDEC
PCIF1
DMRTA1
PRDM16
BLK
SELE
SELL
SELP
SELPLG
SEMA3F
TSPYL2
CDH23
LHPP
TNMD
SLC39A8
FN3K
NOD2
IFIH1
SELENOP
ECSCR
STRA6
ABCG5

ABCG8
SFRP5
SRSF5
SRSF6
FBRS
COP1
IKZF5
SFTPB
HHIP
SFTPC
SFTPD
SGCA
SMIM10L2B
SGK1
SHBG
SHC1
EBF2
GORASP1
PDIA2
WDR13
MFSD1
SI
ST6GAL1
P2RY12
ST3GAL4
GNPNAT1
PMEL
AGXT2
SIX1
BMP2
PINK1
SKP2
SLC1A3
RTN4R
WNK1
SLC2A1
SLC2A2
SLC2A3
SLC2A4
SLC3A1
BMP4
SLC5A1
SLC5A2
SLC5A3

UBE2Z
SLC5A5
SLC6A2
SLC6A3
CXADRP1
SLC6A4
NCF1
SLC6A8
SFTPA1
GGTLC5P
BMP6
SLC7A2
SLC9A1
BMP7
SLC9A3
SLC10A2
SLC11A1
SLC12A3
SLC14A1
SLC34A1
SLC18A2
SLC19A1
SLC20A1
SLC20A2
SLC22A1
SLC22A2
BMPR2
SMARCA4
CYP4F12
SMO
SMPD1
SMS
SNAI1
SNAP25
SNCA
SNRNP70
SOAT1
H3P37
SOD1
SOD2
SOD3
BNIP3L
SORD
SORL1

SOS1
SOX2
SOX4
SOX9
SP1
DST
SPARC
SPG7
SPINK1
SPINT1
SPP1
SPR
SPRR2A
SRD5A2
AKR1D1
BRCA1
SREBF1
SREBF2
SRY
TRIM21
ITPRID2
BRCA2
SST
SSTR4
SSTR5
ST2
ST13
ST14
STAT1
STAT2
STAT3
STAT4
STAT5A
STAT5B
STC1
ELOVL4
STIM1
STK11
BRS3
STX1A
STX4
STXBP1
STXBP3
SULT1A1

BSG
ABCC8
SUV39H1
VAMP2
BTC
SYN2
SYP
SYT1
SYT5
TAC1
ADAM17
TACR1
KLF9
TAGLN
KLF5
TALDO1
TAP1
TAP2
TAPBP
TAT
TBX1
BTF3P11
TBP
TBXA2R
TCF4
HNF1A
HNF1B
TCF3
MIR579
TCF7
MIR615
MIR618
TCF7L2
ZEB1
TCF19
TCF21

TRBV20OR9-2
TRD
BUB1
TDGF1P3
TDO2
TEK
TERC

TERF1
TERT
TFAM
TFE3
TFF3
TFPI
TFRC
TG
TGFA
TGFB1
TGFB2
TGFB3
TGFBI
TGFBR1
TGFBR2
TGM2
TH
THBD
THBS1
THBS2
TSPO
THBS4
THRA
THRB
KLF10
TIE1
TIMP1
TIMP2
TIMP3
TIMP4
C1QBP
TJP1
TKT
TLE1
TLE3
TLR1
TLR2
TLR3
TLR4
TLR5
TSPAN7
TSPAN8
TM7SF2
TMSB4X

TNF
TNFAIP3
TNFRSF1A
TNFRSF1B
TNNI3
TP53
TPD52
C2
TPM4
TPO
C3
CRISP2
NR2C2
TRAF6
C4A
C4B
TRPC3
TRPC6
TRPM2
INS-IGF2
TSC1
TSC2
TSHR
TST
TTC4
TTN
TTPA
TTR
C5AR1
GGTLC3
POTEF
GGT2
OPN1MW2
TWIST1
TNFSF4
CCR2
TXN
TYK2
GGTLC4P
UBE2I
UBE2V1
UCHL1
UCN
C9

UCP1
UCP2
UCP3
UGCG
UMOD
UPP1
UPK2
VPS51
USF1
UTRN
KDM6A
VASP
VCAM1
VDAC1
VDR
VEGFA
VEGFB
VEGFC
VGF
VHL
EZR
VIM
VIP
BEST1
VPREB1
TRPV1
VWF
LAT2
WFS1
NSD2
WNT1
WNT2
WNT5A
WNT6
WNT7A
WRN
XBP1
XBP1P1
XK
XPC
XRCC1
XRCC4
YES1
YY1

YWHAZ
SF1
ZFP36
PTTG1IP
CNBP
CA1
CA2
CACNA1A
ZMYM2
CACNA1E
ZNF236
SLC30A3
DDR1
AIR
PSS
NPHS2
PXDN
ALMS1
TUBA1A
PAX8
CXCR4
FZD5
SCG2
SLMAP
MANF
SLC25A20
CAD
ST8SIA4
ASPSCR1
FTO
NDUFAF5
CCDC28B
MBOAT7
FSD1
DDX39B
CALB1
NOX5
PLA2G7
PAGR1
TNDM
NTT
SLC52A2
CORO7
MUL1

CALCA
ADIPOR2
NAA16
RNASEH2B
AIMP2
USB1
PPP1R3B
DHX40
NLRX1
VTCN1
LIN28A
PALB2
FZD3
ARMC5
SHCBP1
VASH2
EHMT1
HDAC11
CALCR
DCAF17
CALM1
BICC1
NR4A3
ZC3H12A
ASRGL1
MOGAT2
CTC1
ALPK1
NUBPL
STAM
SCUBE1
CDK5RAP3
CUBN
FLAD1
PDGFD
PNPLA3
COASY
CALM2
PDHX
ECB2
THSD7B
FGF23
SPX
COL18A1

CALM3
ZNF436
KMT2D
SETD7
AAAS
HMGA2
SLC2A10
KCNH6
CALR
HBHR
SLC7A5
SLC38A1
TXNDC5
TSC22D4
MAP1LC3B
AKAP1
OR2B2
OR12D3
MED25
MKKS
TAM
CANX
CAPN1
CAPN2
CAPN3
UBL4A
TKTL1
CAPS
CAST
AXIN1
BRAP
FZD4
CARS1
CASP1
ELOVL3
GPR101
MFRP
CASP3
SESN2
CRISPLD2
LOH19CR1
KCNK16
SETDB2
ANGPTL6

FSD1L
MIXL1
PIP5K1B
PLA2G6
CCDC8
PLA2G10
MAGT1
TAGLN2
TKTL2
CASP8
ARL6
SLC41A2
EEA1
RNASEH2C
ASCC2
NLRC5
CASP9
MEHMO
RPAIN
NR0B2
CASQ1
SYVN1
HOPX
CUL1
CASR
ZGPAT
KLF11
KISS1R
SPZ1
TRIM63
ACCS
INSM2
CAT
IRS4
OGT
CNDP1
LMNB2
ATP5MD
HAVCR2
ORAI1
PLXDC2
PPP1R15B
UBASH3B
ELP1

CST7
ABCC11
KRT90P
APOL1
LGR5
MAPKAPK5
GALP
DEGS1
DENR
MADD
CAV1
KHSRP
CAV3
RUNX2
TNFSF11
STC2
PDE8B
TP63
AOC3
ABCB11
NCOA1
SOCS1
PDE5A
DYNLL1
IRS2
VAMP4
BECN1
CBLB
PEA15
IDDM17
DGAT1
RIPK1
HRK
TNFSF14
TNFSF10
CBS
TNFRSF6B
FADD
SIGLEC5
TNFRSF11A
TNFRSF10B
SUCLA2
TRIM24
DPM1

CES2
NRP1
CCN5
CCN4
HDAC3
PROM1
CCK
KAT2B
ASAP2
ALDH1A2
NR1I2
CCKAR
APLN
PER3
CCKBR
CDC123
ARHGEF7
SPHK1
SQSTM1
FUBP1
ZPR1
EIF2S2
WASF1
MGAM
WNT3A
HSPB3
TRPA1
SELENBP1
F2RL3
SOCS3
UCN2
CH25H
BAZ1B
KNSTRN
BMF
RPL14
ARTN
AIP
GPRC5A
SLC7A7
PYGO2
IL33
USP8
MTA1

CD1D
SLC16A4
SLC16A3
ARHGEF1
PDCD5
HGS
UBXN11
PCSK7
CD3E
LPAR2
ARHGEF2
RFT1
TIMD4
NDUFAF2
ARRDC4
SLC33A1
HACD1
CTBP1-DT
GGTLC1
MTG1
CD5L
PTTG1
NAF1
NOG
MSC
CD8A
MFHAS1
STK17B
PIWIL1
DNER
SLC38A5
CD14
GPR55
UBE2Q2
S1PR2
ACVR2B
CD19
MS4A1
KLF4
CD163
B4GALT5
GLP2R
SLIT2
TRMT10A

PPIG
LONP1
KL
ADIPOQ
COX5A
CD27
CIAO1
HS6ST1
CD101
FOXP2
CD28
GRAP2
MUC16
CD80
FADS2
CD86
CYP7B1
NTN1
TP53INP1
ABCG2
NCR1
AIM2
MAP4K4
CD33
EIF2AK3
HOMER1
FHL5
PCYT1B
CHST3
CD34
ROCK2
NAPSA
MAPK8IP1
CD36
SCARB1
ADAMTS4
ADAMTS1
GAL3ST1
GDF15
TBPL1
CD38
NPEPPS
ENTPD1
BAG3

EI24
CIR1
CCL4L2
H6PD
BCAR1
CLOCK
CD40
SOX13
PRDX6
CD40LG
CD44
CARTPT
CD47
ABCG1
IKBKE
SOCS5
CD59
LPIN2
CD68
CD69
NOS1AP
SEMA3E
DCAF1
SART3
HDAC9
HDAC4
ADGRE5
RASSF2
RAPGEF5
PIEZO1
BMS1
IP6K1
GIT2
KEAP1
ELMO1
TBC1D4
NUAK1
RNF10
MFN2
MVP
FGF19
TNFSF15
NR1I3
NR1H4

CCS
CDC42
CDH1
DGCR2
CASP8AP2

HNF4A::GH20J044350

HNF1A::GH12J120977
GPD2

WFS1::GH04J006297

TCF7L2::GH10J113047

TCF7L2::GH10J112997

IGF2BP2::GH03J185795
PRMT7
MIR657
LINC01370
LINC00271

PDZRN3-AS1
WFDC21P
MIR206
MIR125A
NIDDM4
RBPJ
BAIAP2L1
IL6-AS1

LOC110121472
LEPQTL1
NR1H2
G6PC1
LCAT
COG2
PIK3C2A
LPXN
RRAD
APOC2
MT-ND1
PTS

TFAP2B
PNLIP
PDE3B
GRB14
DUSP2
CYP17A1

FTO::GH16J053765
MIR17
MAPK10

KCNQ1::GH11J002824
MIR144
CD63
DRD2
SCT
ICOSLG
SAA1
PDP1
OPRM1
IKBKB
SDHB
GNPDA2
SLPI
MSBP1
SLC27A1
NR1D1
PRKAB2
ELOVL6
KCTD15
RPS27A
METRNL
PLIN5
MTCH2
C1QTNF9
UBE2E2
DLK1
OXT
HMG20A

IGF2R::MIR657 47
SLC47A2
LIPG
MT-CO1

AZGP1
ITGB3
GYS2
ACTRT2
AGMAT
AGRN
AGTRAP
AJAP1
AKR7A2
AKR7A3
ALDH4A1
ANGPTL7
ANIB3
ANON1
ARHGEF10L
ARHGEF16
ATAD3A
ATAD3B
ATP13A2
B3GALT6
BFIS4
CA6
CAMTA1
CAPZB
CCNL2
CCV
CDK11A
CDK11B
CELA2A
CELA2B
CENPS
CEP104
CHD5
CLCN6
CLCNKA
CLCNKB
CLSTN1
CPTP
CROCC
CTNNBIP1
DFFA
DFFB
DFNB96
DHRS3

DISP3
DNAJC11
DVL1
DYT13
EA8
EFHD2
EMC1
EPHA2
ESPN
EXOSC10
FAAP20
FBXO2
FBXO42
FBXO44
FBXO6
GABRD
GBD2
GLC3B
GNB1
GPR153
HES2
HES4
HES5
HSPB7
HTR6
ICMT
INTS11
ISG15
KCNAB2
KIF1B
KLHL17
KLHL21
KONDS
LRRC38
LRRC47
LZIC
MAD2L2
MEGF6
MFAP2
MIB2
MICOS10
MIIP
MIR551A
MMEL1

MMP23A
MMP23B
MRPL20
MXRA8
NADK
NBL1
NBPF1
NECAP2
NMNAT1
NOC2L
NPHP4
OTUD3
PADI2
PADI3
PADI6
PANK4
PAX7
PDPN
PERM1
PEX10
PEX14
PGD
PLA2G2E
PLA2G5
PLCH2
PLEKHG5
PLEKHM2
PLOD1
PRDM2
PRKCZ
PSORS7
PTPRZ2
RCC2
RNF186
RNF207
RNU1-4
RPL22
RSC1A1
SAI1
SAMD11
SCNN1D
SCZD12
SKI
SLC25A33

SLC2A5
SLC2A7
SLC35E2B
SLC45A1
SLC66A1
SMIM1
SPEN
SPSB1
SRARP
SRM
SSU72
TAS1R1
TAS1R2
TAS1R3
THAP3
TMEM240
TNFRSF14
TNFRSF18
TNFRSF25
TNFRSF4
TNFRSF8
TP73
TPRG1L
TRE-TTC3-1
TRG-CCC1-1
UBE4B
UBIAD1
UBR4
VAMP3
VPS13D
VWA1
WRAP73
ZBTB17
ZBTB48
