# Supplementary material for: Network Pharmacology Study to Reveal the Potentiality of a Methanol Extract of Caesalpinia sappan L. Wood against Type-2 Diabetes Mellitus
Source: Life (Basel). 2022 Feb 13;12(2):277. doi: 10.3390/life12020277 (PMC8880704; doi:10.3390/life12020277)
Supplement: Supplementary file 1 [file life-12-00277-s001.zip › Supplementary Table S3.pdf]

Table S3: Common genes between SEA and STP:

Overlapping Genes-238

CA2
SLC13A5
CA1
CA3
CA12
CA14
CA5B
CA13
SI
MAOA
MAOB
CA7
ESR2
CA6
CA4
CA5A
KDM2A
KDM4A
GABRR1
KDM4C
FTO
GAA
ALPL
NAALAD2
ALOX5
MMP9
MMP1
AKR1C3
HSPA1A
AKR1C2
KDM7A
APEX1
ACHE
PTGS2
ODC1
CASP7
SLC22A6
F2
GABBR1
GBA2

CYP1A2
MB
HSD17B3
NQO2
KCNMA1
GPR84
RPS6KA3
HSD17B2
FABP4
TTR
PLAU
MGLL

TUBB1
TLR9
PLA2G10
DHFR
TOP2A
PTPRC
TNKS
CES1
GSTA1
METAP2
ELANE
HTR2A
PRSS1
CYP19A1
CYP11B1
CYP11B2
CYP17A1
TBXAS1
PRKCA
EPHX1
TAAR1
ADRA1A
P2RX7
NAAA
MPO
NR3C1
PGR
VDR
ADH1A
CTSV
ADH1C

XDH
DBH
SNCA
TYR
PDGFRB
NQO1
SRD5A1
SRD5A2
AR
SERPINA6
NR3C2
SHBG
DRD2
HSD11B1
HTR7
TRPV1
FAAH
THRB
DRD3
EPHX2
NPC1L1
PPARA
PPARD
MTNR1A
MTNR1B
HSD11B2
POLB
ACP1
GSR
GLI1
PDE4B
ABCG2
TSPO
BCHE
NR1I2
CDC25A
ADORA3
ESR1
RORA
G6PD
CES2
HMGCR
HSD17B7
NPY5R

CNR1
CRHR1
CYP24A1
FABP3
CYP3A4

ADCY10
ERN1
PPARG
CNR2
NR1H3
CSF1R
RORC
CD38
LYPLA1
LYPLA2
TERT
NR1H4
PTGER4
RARB
PAFAH1B2
APP
PTGFR
GPBAR1
LTB4R
TOP1
CDC25B
PDE4D
ALOX12
PTGER2
PTGIR
OXER1
FFAR4
EDNRA
ENPP2
HNF4A
STS
MPEG1
HCRTR2
DAGLA
PLG
PLAT
ALDH2
CYP27B1

MAPT
AKR1C1
ADH1B
ADH7
AHR
TBXA2R
DHODH
HSD17B1
NOX4
NOX1
EP300
HCRTR1
HTR1A
CFTR
CASP4
CASP9
ITK
AADAT
KYAT3
CYP2C19
TYMP
PHLPP2
ADK
ABCC1
RELA
DNM1
RNPEP
PLA2G2C
S1PR3
SPHK1
VCP
ABCB1
LNPEP
GYS1
HPRT1
PRKCE
BAZ2B
BAZ2A
F2RL3
CBFB
SHH
PNMT
CYP1B1
PTPRS

GLO1
AKR1C4
NEK6
SMARCA2
ST6GAL1

PLA2G2A
POLA1
KCNA3
PTGER3
HSD3B1
GPR18
CISD1
RGS4
KAT2B
GPR55
SREBF2
NR1H2
ASIC3
F7
PARP2
CCR8

GRIK2
NOD1
C1R
