# Supplementary material for: Network Pharmacology Study to Reveal the Potentiality of a Methanol Extract of Caesalpinia sappan L. Wood against Type-2 Diabetes Mellitus
Source: Life (Basel). 2022 Feb 13;12(2):277. doi: 10.3390/life12020277 (PMC8880704; doi:10.3390/life12020277)
Supplement: Supplementary file 1 [file life-12-00277-s001.zip › Supplementary Table S4.pdf]

Table S4: Common genes between T2DM related targets and compound related overlapping genes:

Overlapping genes between CRO and T2DM genes (124 genes)
CA2
CA1
SI
MAOA
CA6
FTO
ALOX5
MMP9
MMP1
HSPA1A
ACHE
PTGS2
ODC1
F2
CYP1A2
MB
KCNMA1
FABP4
TTR
TLR9
PLA2G10
METAP2
ELANE
HTR2A
PRSS1
CYP19A1
CYP11B2
CYP17A1
PRKCA
TAAR1
ADRA1A
P2RX7
NAAA
MPO

NR3C1

VDR
ADH1C
DBH
SNCA
NQO1
SRD5A2
AR
NR3C2
SHBG
DRD2
HSD11B1
TRPV1
THRB
NPC1L1
PPARA
PPARD
MTNR1A
MTNR1B
HSD11B2
ACP1
GSR
ABCG2
TSPO
BCHE
NR1I2
ESR1
G6PD
CES2
HMGCR
HSD17B7
CNR1
CYP24A1
FABP3
CYP3A4
ERN1
PPARG
CNR2
NR1H3
CSF1R
RORC
CD38

LYPLA1
TERT
NR1H4
APP

GPBAR1

LTB4R

PDE4D
ALOX12
OXER1
FFAR4
EDNRA
HNF4A
STS
PLG
PLAT
ALDH2
CYP27B1
MAPT
ADH1B
AHR
TBXA2R
NOX4
NOX1
EP300
HCRTR1
CFTR
CASP9
CYP2C19
ABCC1
RELA
SPHK1
ABCB1
LNPEP
GYS1
F2RL3
GLO1
NEK6
ST6GAL1
PLA2G2A
HSD3B1
CISD1

KAT2B
GPR55
SREBF2
NR1H2
F7
PARP2
NOD1
