# Supplementary material for: Network Pharmacology Study to Reveal the Potentiality of a Methanol Extract of Caesalpinia sappan L. Wood against Type-2 Diabetes Mellitus
Source: Life (Basel). 2022 Feb 13;12(2):277. doi: 10.3390/life12020277 (PMC8880704; doi:10.3390/life12020277)
Supplement: Supplementary file 1 [file life-12-00277-s001.zip › Supplementary Table S5.pdf]

Table S5: Classification of PPI network ordering based on Degree algorithm for 124 common targets

Genes	Degree	Genes	Degree	Genes	Degree
PPARG	36	CASP9	11	SPHK1	6
PTGS2	35	HMGCR	11	ABCC1	6
CYP3A4	35	NR1H3	10	GPBAR1	5
PPARA	33	TRPV1	10	FFAR4	5
ESR1	30	CYP27B1	10	F7	5
EP300	28	CFTR	10	ODC1	5
MMP9	24	GPR55	9	TSPO	5
CYP2C19	24	SHBG	9	ALDH2	5
CYP19A1	21	PLA2G10	9	ADH1B	5
AHR	21	HTR2A	9	CA1	5
AR	21	NOX4	8	F2RL3	4
NR3C1	21	PPARD	8	MTNR1A	4
CYP1A2	20	FABP4	8	NAAA	4
CYP17A1	19	NR1H2	8	CD38	4
RELA	19	MMP1	8	KCNMA1	4
MPO	18	TLR9	8	LTB4R	4
HSPA1A	17	SRD5A2	8	GLO1	4
APP	17	NR3C2	8	ACP1	4
HNF4A	17	STS	8	CES2	4
ABCB1	17	PLA2G2A	8	P2RX7	3
F2	16	VDR	8	FTO	3
PLG	16	NOX1	8	THRB	3
MAOA	15	MAPT	8	CSF1R	3
CNR1	15	TERT	7	CISD1	3
HSD3B1	14	HSD11B2	7	ERN1	3
GSR	14	CYP11B2	7	ADH1C	3
NR1H4	14	CNR2	7	METAP2	2
NR1I2	14	ELANE	7	LYPLA1	2
ABCG2	14	MB	7	GYS1	2
PRKCA	13	PRSS1	7	HCRTR1	2
NQO1	13	KAT2B	7	TAAR1	2
HSD11B1	12	NPC1L1	6	NOD1	2
ALOX5	12	FABP3	6	RORC	2
ACHE	12	HSD17B7	6	LNPEP	1
DRD2	12	MTNR1B	6	EDNRA	1
SREBF2	11	CA2	6	PARP2	1
BCHE	11	PLAT	6	CA6	1
TTR	11	ALOX12	6	OXER1	1

G6PD	11	TBXA2R	6	ADRA1A	1
SNCA	11	SI	6		
CYP24A1	11	DBH	6		

Table S5.1: Substances are categorized in the Compound-Targets network according to degree value of importance

Rank	Compounds	Degree	Rank	Compounds	Degree
1	Fisetin tetramethyl ether	27	18	Z,Z-10,12-Hexadecadien-1-ol acetate	3
2	9,12-Octadecadienoic Acid (Z,Z)-	25	19	4-phenylpyrido[2,3-d]pyrimidine	2
3	2,4-Dimethoxybenzyl alcohol	24	20	Benzo[h]quinoline, 2,4-dimethyl-	1
4	Clionasterol	20	21	4-Cyclohexene-1,2-dicarboximide, N-butyl-, cis-	1
5	Cholest-4-En-3-One	19	22	6-Amino-5-cyano-4-(5-cyano-2,4-dimethyl-1H-pyrrol-3-yl)-2-methyl-4H-pyran-3-carboxylic acid ethyl ester	1
6	Tetramethylkaempferol	18	23	2-adamantanyl-N-(adamantanylethyl)acetamide	1
7	9,12-Octadecadienoic acid, methyl ester	18	24	2(1H)-Naphthalenone, octahydro-4a-methyl-7-(1-methylethyl)-, (4a.alpha.,7.beta.,8a.beta.)-	1
8	1,3-Benzenediol, Monoacetate	12	25	3-Methyl-2-Pentyl-2-Cyclopenten-1-One	1
9	Neozepam	10	26	Ethyl 2-Methylpropanoate	1
10	Hydroquinone	8	27	2-(4-methylphenyl) Indolizine	0
11	Cholestane, 3,4-epoxy-2-methyl-, (2.alpha.,3.alpha.,4.alpha.,5.alpha.)-	7	27	2-[(4E)-4-Hexenyl]-6-nitrocyclohexanone dimethylhydrazone	0
12	Dehydrodiisoeugenol	7	27	Disalicylalpropylenediimine	0
13	Tetradecanoic Acid, 12-Methyl-, Methyl Ester	7	27	1,3-Dimethyl-5,6-dicarbethoxy-5,6,7,8-tetrahydro-6,7-diazalumazine	0
14	Indole-2-one, 2,3-dihydro-N-hydroxy-4-methoxy-3,3-dimethyl-	6	27	Nadolol di-methylboronic acid	0

15	1,3-Benzenediol	5	27	Aspidodispermine, O-methyl-	0
16	5-methyl-2-phenyl- 1H-Indole	4	27	(3E)-5-Hydroxy-2-methyl-3-hexenoic acid	0
17	Propenone, 1-[5-(3-hydroxy-3-methyl-1-butynyl)-2-thienyl]-3-(4-methoxyphenyl)-	4			

Table S5.2: Enrichment of T2DM-associated common target genes in 9 KEGG pathways

Terms	Genes	Q-value
hsa03320:PPAR signaling pathway	FABP3, FABP4, MMP1, NR1H3, PPARG, PPARA, PPARD	0.002672
hsa04976:Bile secretion	ABCB1, CA2, NR1H4, HMGCR, CFTR, ABCG2	0.014743
hsa05200:Pathways in cancer	CSF1R, MMP1, PRKCA, PTGS2, MMP9, RELA, CASP9, AR, EDNRA, EP300, PPARG, F2RL3, PPARD	0.077293
hsa04020:Calcium signaling pathway	P2RX7, EDNRA, TBXA2R, SPHK1, CD38, PRKCA, HTR2A, ADRA1A	0.114384
hsa01100:Metabolic pathways	ADH1C, MAOA, ADH1B, ODC1, ALOX12, HMGCR, DBH, CYP2C19, HSD17B7, CYP3A4, PTGS2, CYP19A1, CYP17A1, HSD11B1, CYP27B1, ALDH2, SI, ALOX5, CD38, ST6GAL1, G6PD, SPHK1, PLA2G2A, HSD3B1, CYP24A1, CYP11B2, CYP1A2, PLA2G10	0.165218
hsa04919:Thyroid hormone signaling pathway	CASP9, KAT2B, THRB, EP300, PRKCA, ESR1	0.202243
hsa04152:AMPK signaling pathway	GYS1, HNF4A, PPARG, HMGCR, ADRA1A, CFTR	0.261061
hsa05202:Transcriptional misregulation in cancer	CSF1R, PPARG, PLAT, MPO, MMP9, RELA, ELANE	0.303411
hsa05215:Prostate cancer	CASP9, AR, SRD5A2, EP300, RELA	0.351174

Table S5.3: Concrete information on the chemical interactions of key substance with the genes involved in PPAR signaling pathway

Gene	Ligand-Protein complex	Binding Interaction		
		Hydrogen Bond	Hydrophobic interactions	Others
PPARG	3E00 - Fisetin Tetramethyl Ether	ARG-288, SER-342, ILE-262, GLY-258, LEU-340, GLU-259 (11)	ARG-288, ILE-249, LEU-333, LEU-255, ARG-280, ILE-281, CYS-285, ILE-341 (12)	-

PPARA	1K7L - Fisetin Tetramethyl Ether	ASN-219, GLU-286, TYR-334, CYS-278, ILE-317, MET-220 (7)	TYR-334, ALA-333, CYS-278, ILE-317, LEU-321 (7)	MET-320 (1) Pi-Sulfur
PPARD	5U3Q - Fisetin Tetramethyl Ether	MET-192, ALA-306, ASN-307, PHE-190, GLU-259, LEU-304, ILE-290 (8)	LEU-304, ILE-290, LEU-294, LYS-229 (5)	GLU-259 (Electrostatic) (1), MET-293 (Pi- Sulfur) (1)
FABP3	5HZ9 - Fisetin Tetramethyl Ether	GLU-62, THR-61, THR-74, THR-75, ASN-60, ASP-72, ASP-78 (9)	-	LYS-59, ASP-78 (Electrostatic) (2)
FABP4	3P6D - Fisetin Tetramethyl Ether	GLU-61, THR-60, ASN-59, VAL-73, ASP-71 (9)	VAL-73 (3)	-
MMP1	1SU3 - Fisetin Tetramethyl Ether	HOH-951, GLN-50, SER-172, PRO-173, GLU-39, LYS-36, ASN-43 (8)	LYS-36, LYS-40, PRO-95 (6)	-
NR1H3	1UHL - Fisetin Tetramethyl Ether	LYS-317, THR-314, PHE-315, SER-228 (5)	ARG-305, ARG-226, PHE-315, LYS-317 (6)	-
